# Optogenetic modulation of cardiac action potential properties may prevent arrhythmogenesis in short and long QT syndromes

**DOI:** 10.1172/jci.insight.147470

**Published:** 2021-06-08

**Authors:** Amit Gruber, Oded Edri, Irit Huber, Gil Arbel, Amira Gepstein, Assad Shiti, Naim Shaheen, Snizhana Chorna, Michal Landesberg, Lior Gepstein

**Affiliations:** 1 Sohnis Research Laboratory for Cardiac Electrophysiology and Regenerative Medicine, the Rappaport Faculty of Medicine and Research Institute, Technion-Israel Institute of Technology, Haifa, Israel, Haifa, Israel.; 2 Cardiology Department, Rambam Health Care Campus, Haifa, Israel.

**Keywords:** Cardiology, Stem cells, Arrhythmias, Gene therapy, iPS cells

## Abstract

Abnormal action potential (AP) properties, as occurs in long or short QT syndromes (LQTS and SQTS, respectively), can cause life-threatening arrhythmias. Optogenetics strategies, utilizing light-sensitive proteins, have emerged as experimental platforms for cardiac pacing, resynchronization, and defibrillation. We tested the hypothesis that similar optogenetic tools can modulate the cardiomyocyte’s AP properties, as a potentially novel antiarrhythmic strategy. Healthy control and LQTS/SQTS patient–specific human induced pluripotent stem cell–derived cardiomyocytes (hiPSC-CMs) were transduced to express the light-sensitive cationic channel channelrhodopsin-2 (ChR2) or the anionic-selective opsin, ACR2. Detailed patch-clamp, confocal-microscopy, and optical mapping studies evaluated the ability of spatiotemporally defined optogenetic protocols to modulate AP properties and prevent arrhythmogenesis in the hiPSC-CMs cell/tissue models. Depending on illumination timing, light-induced ChR2 activation induced robust prolongation or mild shortening of AP duration (APD), while ACR2 activation allowed effective APD shortening. Fine-tuning these approaches allowed for the normalization of pathological AP properties and suppression of arrhythmogenicity in the LQTS/SQTS hiPSC-CM cellular models. We next established a SQTS–hiPSC-CMs–based tissue model of reentrant-arrhythmias using optogenetic cross-field stimulation. An APD-modulating optogenetic protocol was then designed to dynamically prolong APD of the propagating wavefront, completely preventing arrhythmogenesis in this model. This work highlights the potential of optogenetics in studying repolarization abnormalities and in developing novel antiarrhythmic therapies.

## Graphical abstract

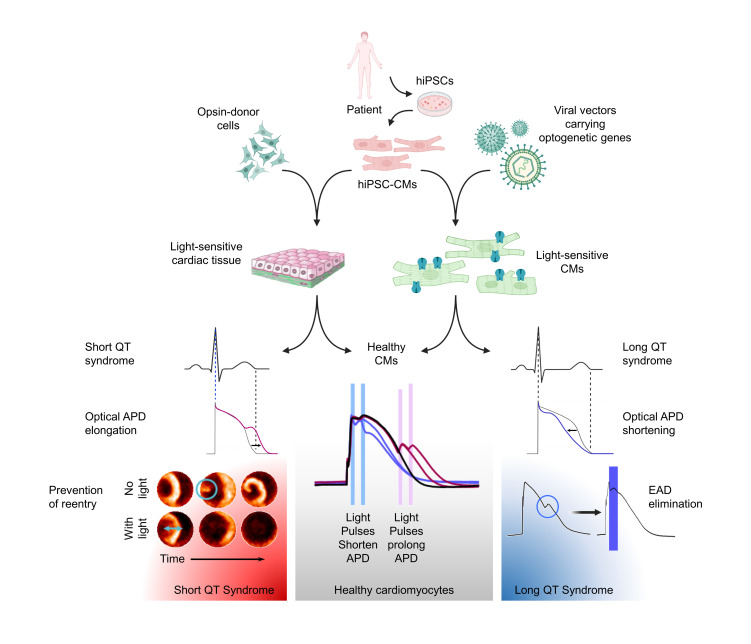

## Introduction

The cardiac action potential (AP) results from a balanced integration of several distinct ionic currents (1), and when disrupted, it could lead to life-threatening arrhythmias. Long and short QT syndromes (LQTS and SQTS, respectively) are examples of inherited arrhythmogenic syndromes, where mutations in ion channels can lead to abnormal AP duration (APD) prolongation or shortening, respectively (2–4). When the APD is too long, spontaneous early-after-depolarizations (EADs), triggered beats, and life-threatening ventricular arrhythmias can occur. When APD is abbreviated, the refractory period and tissue wavelength (WL) are shortened, increasing the risk for reentrant arrhythmias (5).

Optogenetics allows to control neuronal activity through the expression of light-sensitive microbial proteins (opsins), functioning as ion channels or pumps (6–8). More recently, similar concepts were applied to the heart (9–12), leading to development of experimental optogenetic-based cardiac pacing (13, 14), resynchronization (14, 15), and defibrillation (16–20) approaches. While the focus of cardiac optogenetics has been on inducing or suppressing AP generation with depolarizing or hyperpolarizing light-sensitive proteins, similar concepts could potentially be used to modulate AP properties, as suggested by computational modeling (21), proof-of-concept experiments using optical dynamic clamp studies for drug testing in human cardiomyocytes (CMs) (22), and studies using neonatal rat CMs (23, 24).

Here, we aimed to take this concept a step forward by evaluating the ability to optogenetically shape the AP morphology of human CMs and, specifically, those displaying clinically relevant abnormalities. To this end, we used healthy control and patient-specific SQTS and LQTS induced pluripotent stem cell–derived CMs (hiPSC-CMs) that were either genetically modified to express light-sensitive channels or cocultured with opsin-expressing engineered cells. By activating the nonselective cationic light-sensitive channel channelrhodopsin-2 (ChR2), we were able to induce both shortening and robust prolongation of APD, depending on illumination timing. Light activation of the anionic-selective opsin anion channelrhodopsin 2 (ACR2) allowed for more effective APD shortening. Importantly, by using such optogenetic protocols, we could normalize the pathological AP properties and reduce arrhythmogenicity in the patient-specific LQTS/SQTS hiPSC-CM at the cellular levels. Finally, using a unique tissue model of reentrant arrhythmias, derived using the SQTS–hiPSC-CMs, we were able to demonstrate the ability to prolong and correct APD also at the tissue level and, importantly, to design and pattern dynamic optogenetic AP-modulating protocols that can completely prevent arrhythmogenesis.

## Results

### Characterization of ChR2-expressing hiPSC-CMs.

**Figure 1:**
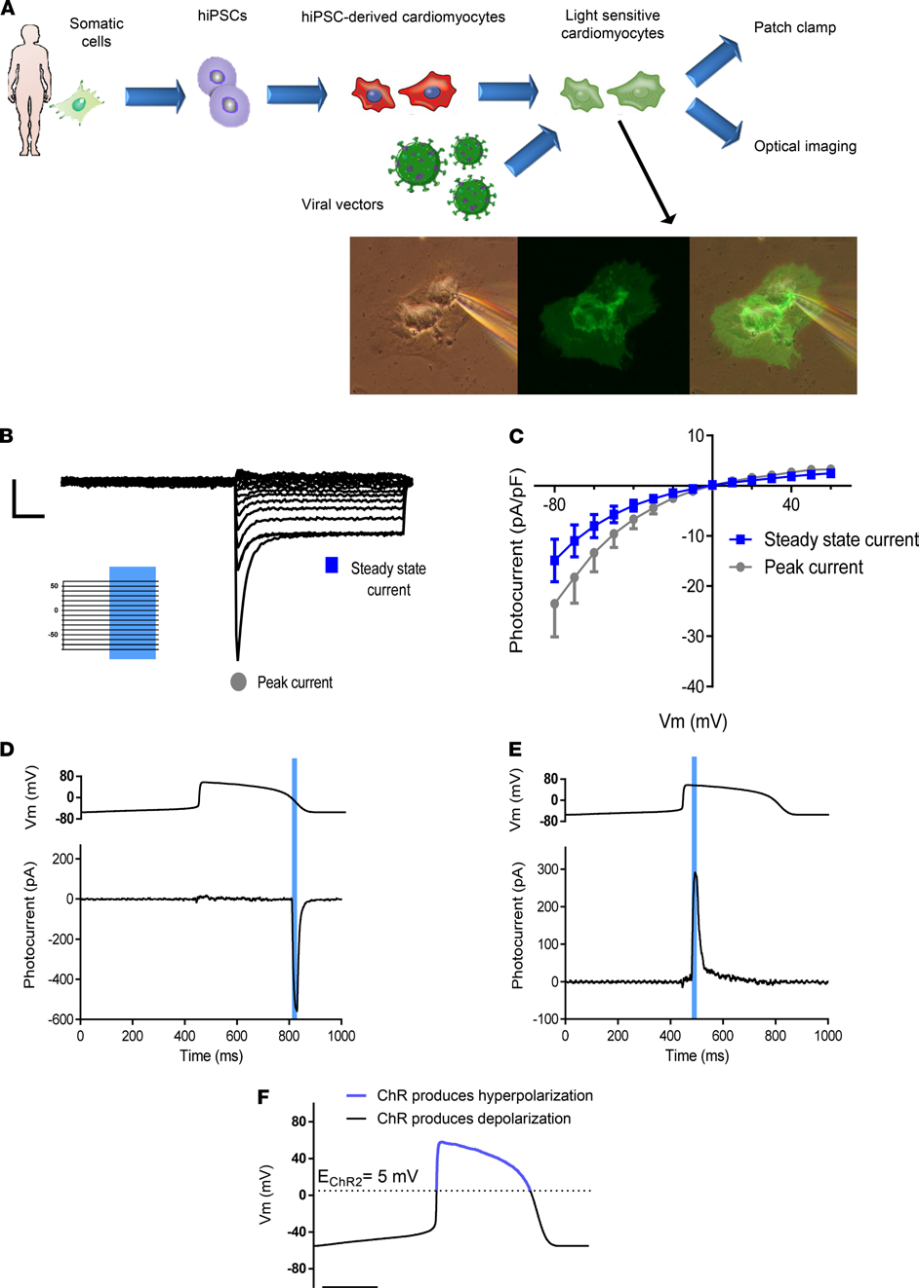
Experimental scheme and functional characterization of ChR2 photocurrents in hiPSC-CMs. (**A**) Experimental outline: patient-specific or control hiPSCs are differentiated into cardiomyocytes, transduced to express the different opsins, and subjected to patch-clamp or optical imaging analysis. Total original magnification, ×28. (**B**) Representative traces showing 1 example of 7 similar voltage-clamp experiments in the ChR2-expressing hiPSC-CMs. Peak and steady-state photocurrents are shown. Scale bars: 100 pA and 100 ms on *y* and *x* axes, respectively. The stimulation protocol (insert) included voltage steps of 1 second (first 500 ms conducted in darkness followed by 500 ms of continuous blue light illumination) from –80 mV to 60 mV, with 10 mV increments. (**C**) Current-voltage relationship of the ChR2 photocurrents. Mean ± SEM of peak and steady state currents are plotted (*n =* 7). (**D** and **E**) Representative traces (from 5 experiments) describing the photocurrents evoked in the hiPSC-CMs by light-stimuli applied during phase 2 (**D**) or 3 (**E**) in the AP clamp experiments. The upper panel shows the voltage AP clamp protocol, while the lower panel depicts the measured photocurrents [after baseline (darkness) subtraction]. (**F**) The conceptual differences in the type of photocurrents generated by light-induced ChR2 activation during different AP phases. An optical stimulus will produce a hyperpolarizing current if the Vm is more positive than ChR2-E_Rev_ (early phase 2); whereas a depolarizing current will be generated if Vm is more negative than ChR2-E_Rev_ (repolarization phase). Scale bar: 200 ms.

CM differentiation of healthy control hiPSCs (25, 26) was achieved using a monolayer-based directed differentiation system (27, 28). The generated hiPSC-CMs were dispersed into single cells, transduced to express the ChR2-eGFP transgene, and evaluated by patch-clamp or optical imaging experiments (Figure 1A). Whole-cell voltage-clamp experiments performed during darkness and under blue light illumination (470 nm monochromatic-light, 1.3 mW/mm^2^) identified a robust light-sensitive current in the ChR2-expressing hiPSC-CMs (Figure 1, B and C). The resulting current-voltage curves (Figure 1C) revealed the typical inward rectification properties of ChR2 (29) with a reversal potential (ChR2-E_Rev_) of approximately 0 mV.

We next evaluated the ability to produce photocurrents in the hiPSC-CMs during the AP by predesigned, time-targeted, illumination protocols. To this end, we designed an AP clamp experiment and timed the optical stimulus either during the repolarization phase of the AP (phase 3) when the membrane potential (V_m_) becomes relatively negative (Figure 1D) or during early phase 2, when V_m_ is relatively positive (Figure 1E). Interestingly, light-induced ChR2 activation during phase 3 resulted in a robust inward depolarizing current (Figure 1D), whereas early phase 2 stimulation produced an outward hyperpolarizing photocurrent (Figure 1E).

The mechanism underlying this surprising difference stems from the relationship between ChR2-E_Rev_ and V_m_ (Figure 1F). When V_m_ is more negative than ChR2-E_Rev_ (late repolarization), an optical stimulus will cause a depolarizing current, whereas it will produce a hyperpolarizing current if V_m_ is more positive than ChR2-E_Rev_ (during early phase 2).

### Optogenetic AP modulation in ChR2-expressing hiPSC-CMs.

**Figure 2:**
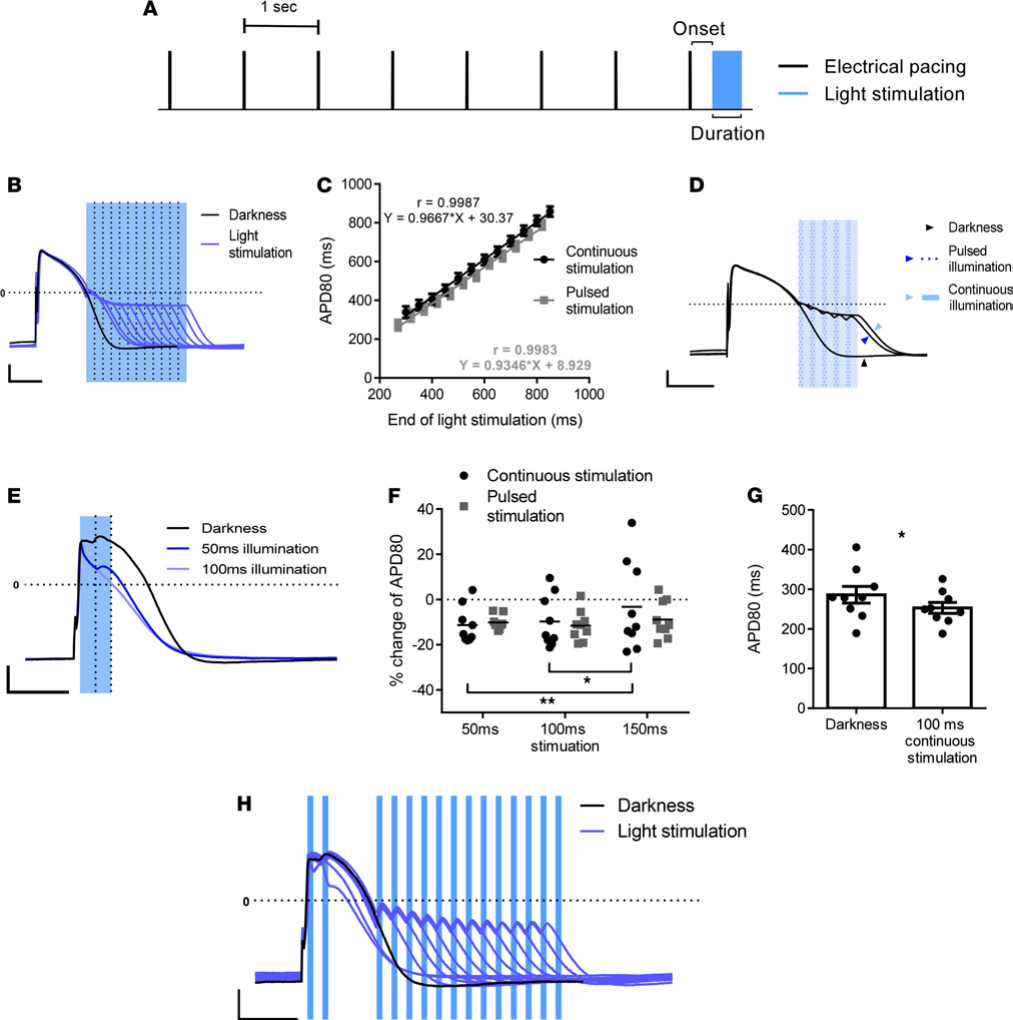
Optogenetic APD modulation in ChR2-expressing hiPSC-CMs. (**A**) Optogenetic protocols included 7 electrically stimulated APs at 1 Hz with delivery of optogenetic stimuli at a specific timing following the last electrical pacing stimulation (onset). (**B**–**D**) Light-induced ChR2 activation during phase 3 prolongs APD in whole-cell current clamp recordings. (**B**) Representative AP traces acquired during darkness (black) and following different optogenetic stimuli (blue) of various durations (dashed lines). Note the tight correlation between optical stimulus duration and the resulting APD prolongation. (**C**) A plot depicting the correlation between the timings of the end of the optical stimuli and the resulting APD_80_ values. Both continuous (black circles) and pulsed (gray squares) illumination protocols resulted in high correlations (*r* = 0.99 and 0.99, *n* = 12; regression models are presented). (**D**) Comparison of continuous and pulsed (20 ms on/30 ms off) illumination effects showing similar APD prolongations. (**E**–**G**) Early light-induced ChR2 activation shortens APD. (**E**) Representative AP traces from 9 experiments acquired during darkness (black) and with early optical stimuli of various durations (onset, 20 ms). (**F**) Comparing the effects achieved by varying optical stimulus durations (50 ms, 100 ms, and 150 ms, *n* = 9) on the relative APD_80_ shortening using both continuous (black) and pulsed (gray) stimulation protocols. Note that, due to the limited time window for APD shortening, the longest continuous illumination tested (150 ms) is significantly less. *P < 0.05 and **P < 0.01 using 2-way ANOVA test for repeated measurements, followed by post-hoc Tukey test. (**G**) Shortening of the measured APD_80_ values following early optogenetic stimulation (onset, 20 ms; duration, 100 ms) (*P < 0.05 using paired Student’s *t* test, *n* = 9). (**H**) Summary of the bidirectional APD modulating effects of ChR2 light activation as function of the timing and duration of the optical stimulus. Scale bars: 20 mV and 200 ms for the *y* and *x* axes, respectively (**B**, **D**, **E**, and **H**).

We next aimed to utilize the ChR2-induced depolarizing or hyperpolarizing photocurrents to modulate the AP properties of hiPSC-CM. To this end, hiPSC-CMs were paced at 1 Hz for 7 seconds (at darkness) and then subjected to different illumination protocols delivered during the last AP (Figure 2A). Initially, we synchronized illumination to phase 3 of the AP to trigger ChR2-related depolarizing currents and noted, by whole-cell recordings, marked APD prolongation (Figure 2B). Interestingly, the degree of APD prolongation highly correlated (*r* = 0.99, *n =* 12, Figure 2C) with illumination duration (Figure 2, A–C).

We also demonstrated that the continuous illumination protocol could be replaced by a pulsed stimulation protocol (repeated cycles of 20 ms light-on/30 ms light-off). This pulsed illumination protocol resulted in comparable APD_80_ (time to 80% repolarization) prolongations (Figure 2, C and D), which also correlated with the total stimulus duration (*r* = 0.99, *n =* 12, Figure 2C). The importance of this pulsed-protocol is 2-fold; it may allow simultaneous illumination and optical AP recording by reducing illumination-related artifacts (see below), and it may also reduce energy requirements.

To evaluate the degree of illumination time reduction that can be achieved using the pulsed illumination protocol, which still resulted in comparable functional outcome, we tested 11 different protocols of delivering 1 second–long pulsed (20 Hz) optical stimulations. The duty cycles tested (representing the percentage of the actual illumination time) ranged from 100% (continuous illumination) to 80%, 60%, 40%, 20%, 10%, 4%, 2%, 1%, 0.2%, and 0.1%. As seen in Supplemental Figure 1 (supplemental material available online with this article; https://doi.org/10.1172/jci.insight.147470DS1), quantifying the changes in AP morphology, plateau V_m_, and oscillations’ amplitudes, one could reduce the actual illumination time exposure to very low values (1%) without altering the functional outcome.

We next tested the hypothesis that ChR2 activation can also be used for APD shortening by synchronizing illumination to early phase 2 (Figure 2E), when V_m_ is more positive than ChR2-E_Rev_. The durations of the tested optogenetic stimuli (onset, 20 ms), delivered either as continuous or pulsed (20 ms on/30 ms off) illumination were 50 ms, 100 ms, and 150 ms. These illumination protocols resulted in APD_80_ shortening by approximately 10%–15% (Figure 2, E and F). For example, at illumination duration of 100 ms, APD_80_ was shortened from 286 ± 21 ms to 253 ± 4 ms (*P <* 0.05, *n =* 9, Figure 2G). Interestingly, illumination durations of 50 and 100 ms were early and short enough to activate the cells only when their V_m_ were more positive than the ChR2-E_Rev_ (>0 mV) and, therefore, only shortened APD (Figure 2E). In contrast, illumination durations that exceed this time window for ChR2-mediated APD shortening (such as 150 ms illumination) were less potent for that purpose and could even prolong APD at the time when V_m_ is lower than ChR2-E_Rev_ (Figure 2F).

Taken together, our results, summarized in Figure 2H, highlight how light-induced ChR2 activation can be utilized to bidirectionally affect AP properties, either prolonging or shortening APD, depending on illumination timing.

### Optogenetic APD modulation in LQTS and SQTS hiPSC-CMs.

We next aimed to use the optogenetic strategies described above to modulate AP properties in pathological states. To this end, we utilized previously established patient-specific LQTS (25) and SQTS (30) hiPSC-CM models. In LQTS2, loss-of-function *KCNH2* mutations reduce the rapid component of the delayed-rectifier potassium current (I_Kr_) thereby prolonging APD, whereas in SQTS1, gain-of-function mutations in the same gene augments I_Kr_, leading to APD shortening.

**Figure 3:**
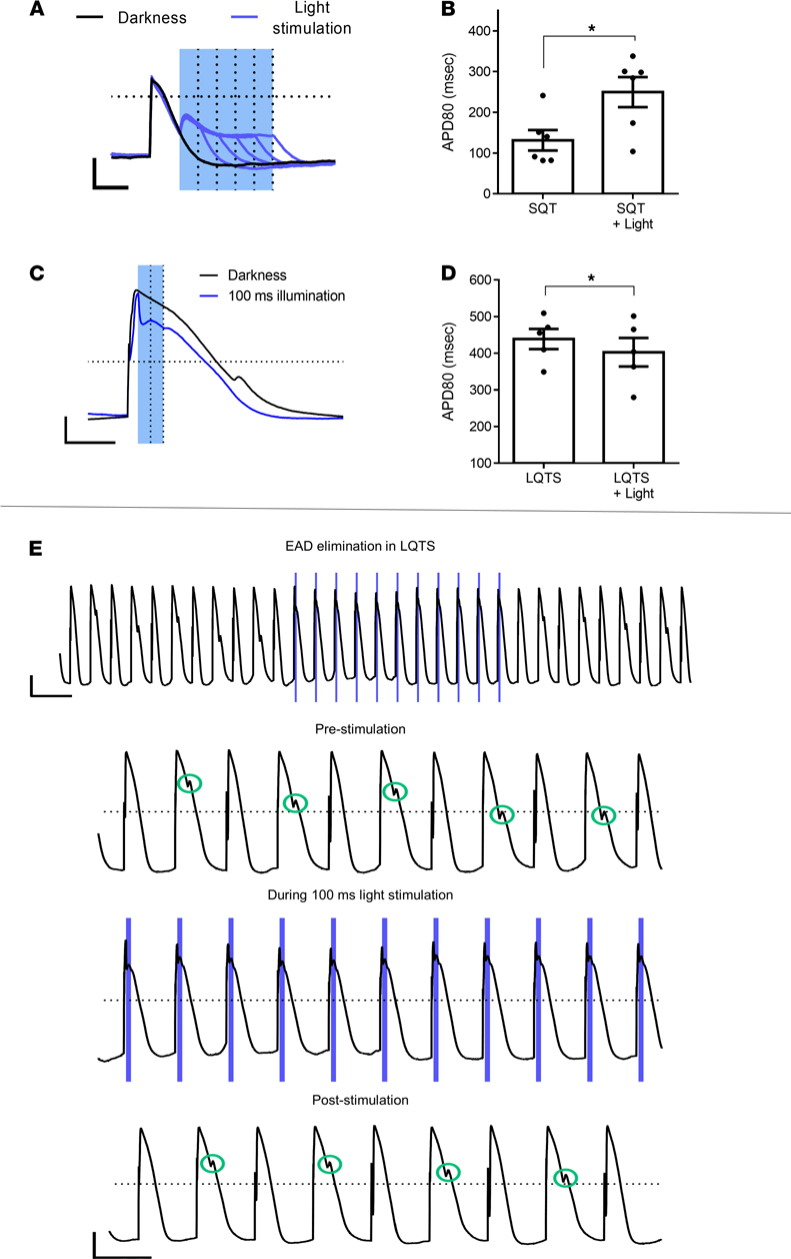
Optogenetic APD modulation in ChR2-expressing LQTS– and SQTS–hiPSC-CMs. (**A** and **B**) Light-induced ChR2 activation during the repolarization phase prolonged APD of SQTS–hiPSC-CMs with the degree of APD prolongation correlating with illumination duration (**A**). Application of a 250 ms–long optical stimulus (onset = 80 ms) was able to significantly prolong APD_80_ (*n =* 6, *P <* 0.05 using paired *t* test) in the SQTS–hiPSC-CMs (**B**). (**C** and **D**) Light-induced ChR2 activation, early during the AP, could shorten the abnormally long APD of the LQTS–hiPSC-CMs (**C**). Scale bars: (**A** and **C**) 20 mV and 200 ms for the *y* and *x* axes, respectively. The degree of APD_80_ shortening achieved by the optimal stimulation protocol (onset, 40 ms; duration, 100 ms) was statistically significant (*P < 0.05, *n =* 5) using paired *t* test (**D**). (**E**) Light-induced ChR2 activation during early phase 2 of the AP phase suppresses EAD formation in LQTS–hiPSC-CMs. Shown are AP recordings from the LQTS–hiPSC-CMs at baseline (1 Hz electrical pacing), during the application of the illumination protocol (onset, 40 ms; duration, 100 ms) for each individual AP and following termination of illumination. The lower 3 panels present higher time resolution of the upper panel, showing the development of EADs at baseline in some paced beats; the suppression of EADs in all APs following illumination (blue lines); and resumption of arrhythmogenic activity following illumination termination. EADs are highlighted with green circles. Scale bars: 20 mV for the *y* axis, 2 seconds for the *x* axis of the upper panel, and 1 second for the 3 lower panels.

Lentiviral transduction was used to express ChR2 in the SQTS– and LQTS–hiPSC-CMs. Similar to results in healthy hiPSC-CMs (Figure 2, B–D), light-induced ChR2 activation during repolarization markedly prolonged APD_80_ in the SQTS–hiPSC-CM, with the degree of APD prolongation correlating with optical signal duration (Figure 3A). Consequentially, one could tailor optical stimulation to achieve any desired APD value. For example, application of a 250 ms–long stimulus was able to significantly prolong the APD_80_ values in the SQTS–hiPSC-CMs from 132 ± 25 ms to 250 ± 37 ms (*n =* 6, *P <* 0.05, Figure 3B). Comparing APD_80_ values in the untreated and optogenetically treated ChR2-expressing SQTS–hiPSC-CMs with healthy control hiPSC-CMs (283 ± 19 ms, *n =* 10, Supplemental Figure 2A), we noted that, while untreated SQTS–hiPSC-CMs displayed significantly (*P <* 0.001) shorter values than healthy control cells, optogenetic treatment was able to fully correct APD values to levels that were not statistically different than control CMs.

We next tested the hypothesis that light-induced ChR2 activation during early phase 2 could shorten APD in the LQTS–hiPSC-CMs. Similar to healthy hiPSC-CMs (Figure 2, E–G), an optogenetic stimulus delivered during early phase 2 (onset, 40 ms; duration, 100 ms) was able to significantly shorten the abnormally long APD values in the LQTS–hiPSC-CMs (*P <* 0.05, *n =* 5, Figure 3, C and D). However, in contrast to the unrestricted APD prolongation by late ChR2 activation in SQTS–hiPSC-CMs, the maximal degree of APD shortening achieved by early ChR2 activation in LQTS–hiPSC-CMs was limited (Figure 3, C and D). Thus, when comparing APD_80_ values in the untreated and optogenetically treated ChR2-expressing LQTS–hiPSC-CMs with healthy control hiPSC-CMs, we noted that, despite the APD shortening achieved by optogenetics modulation, it was still not sufficient to reach the levels in healthy control hiPSC-CMs (*P <* 0.01; *n =* 5 and 10 for the LQTS and healthy control hiPSC-CMs, respectively; Supplemental Figure 2B).

Despite the less than optimal APD shortening, the effect achieved by ChR2 activation was still sufficient to provide an antiarrhythmic benefit. This was evident by studying LQTS–hiPSC-CMs that also displayed EADs, the harbinger of arrhythmias in LQTS (4). Such EAD-displaying LQTS–hiPSC-CMs during continuous 1 Hz electrical pacing (Figure 3E) were subjected to 100 ms–long (onset, 40 ms) optical stimuli, which completely suppressed EAD formation. EADs reappeared immediately after cessation of light stimulation.

We also confirmed that the illumination protocol used to shorten APD and prevent EADs in the ChR2-expressing LQTS–hiPSC-CMs did not affect other component of the AP. As shown in Supplemental Figure 3, application of this optogenetic protocol did not cause any significant changes in the AP’s amplitude, maximum rise slope, or maximal diastolic potential.

### Optogenetic AP modulation using the anion channelrhodopsin-2 (ACR2).

Given the relative limited ability of ChR2 to shorten APD, we hypothesized that a different opsin variant, ACR2 (31), could produce more potent hyperpolarizing effects due to its anionic selectivity, primarily conducting chloride anions. To provide insights into ACR2 photocurrents, we conducted patch-clamp experiments in ACR2-expressing HEK293 cells (Supplemental Figure 4). By using a fixed extracellular [Cl^–^] concentration but varying intracellular [Cl^–^] levels, we confirmed the significance of the transmembrane [Cl^–^] concentration gradient in determining ACR2-E_Rev_ and the resulting photocurrent magnitude. Although intracellular [Cl^–^] levels may vary between different cell types and are difficult to measure (32, 33), they are typically lower than their corresponding extracellular levels, resulting in negative ACR2-E_Rev_ values (Supplemental Figure 4B). Consequentially, light-induced ACR2 activation should induce hyperpolarizing photocurrents throughout the CM’s AP (Supplemental Figure 4C).

**Figure 4:**
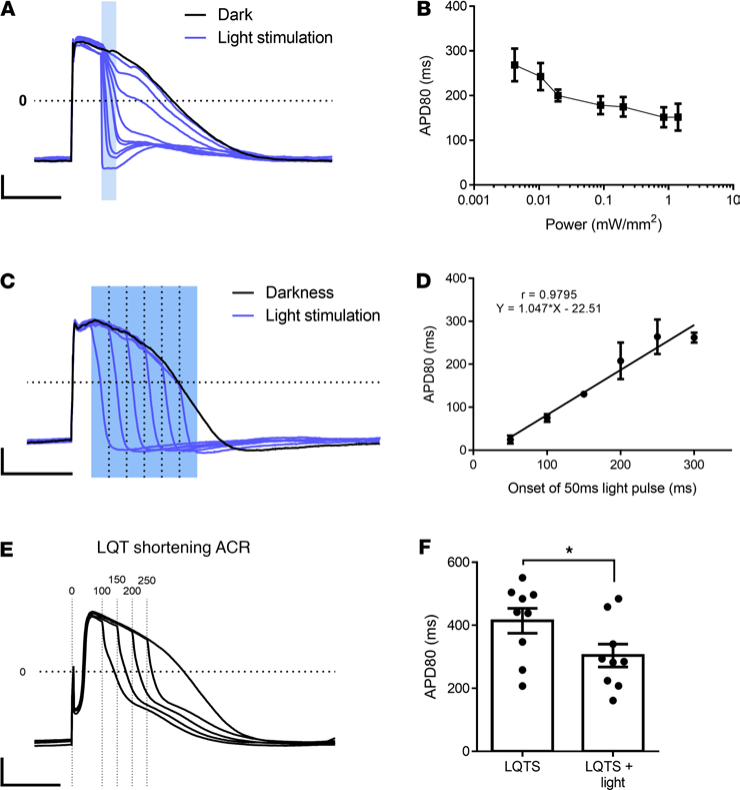
Optogenetic APD modulation in ACR2-expressing hiPSC-CMs. (**A** and **B**) Intracellular recordings (**A**) and a summary plot (**B**) characterizing the changes in AP morphology and APD_80_ values of the ACR2–hiPSC-CMs as a function of the optical stimulus’ intensity (signal duration, 50ms; onset, 100ms). Scale bars: 20 mV and 200 ms for the *y* and *x* axes, respectively; *n =* 5. (**C** and **D**) Intracellular recordings (**C**) and a summary plot (**D**) characterizing changes in AP morphology and APD_80_ values of the ACR2–hiPSC-CMs as a function of the timing of the delivered stimulus (intensity, 1.3 mW/mm^2^; duration, 50 ms) onset. Note the clear correlation (Pearson’s correlation coefficient = 0.98, *n =* 5) between stimulus onset and APD_80_ shortening, with earlier onsets leading to shorter APD_80_ values. (**E**) Patch-clamp recordings showing robust shortening of the abnormally long APD values in LQTS–hiPSC-CMs following light-induced ACR2 activation. APD shortening inversely correlated with the onset of the optical stimulus (intensity, 1.3 mW/mm^2^; duration, 50 ms), which was initiated at 50, 100, 150, 200, or 250 ms after AP onset. (**F**) Summary of the effects of application of a 50 ms–long stimulus (intensity, 1.3 mW/mm^2^; onset, 100 ms) to the ACR2-expressing LQTS–hiPSC-CMs. Notice the significant shortening of APD_80_ values (*n =* 9, *P < 0.01 using paired *t* test).

To confirm the aforementioned assumption and to test the ability of light-induced ACR2 currents to shorten APD, we used lentiviral transduction to express ACR2 in hiPSC-CMs. We then performed patch-clamp studies using an intracellular [Cl^–^] concentration of 4 mM, as reported for neonatal rat CMs (23). As expected, exposure of the electrically paced ACR2–hiPSC-CMs (1 Hz) to different light-stimulation protocols, applied during early phase 2, led to marked APD shortening (Figure 4, A–D). Evaluating the effects of different illumination parameters revealed that higher light intensities (Figure 4, A and B) and earlier stimulus-onset timings (Figure 4, C and D) resulted in greater APD_80_ shortening, allowing the precise control of the CM’s APD. Interestingly, in contrast to the dual effect of ChR2, ACR2 activation could not be used to prolong APD (by inducing depolarizing currents) even when given late during the repolarization phase (Figure 4, C and D).

We next expressed ACR2 in LQTS–hiPSC-CMs and demonstrated the ability to optogenetically shorten and normalize APD values also in pathological states (Figure 4E). The degree of APD shortening inversely correlated with illumination onset (Figure 4E), allowing for the tailoring of APD values to any desired value. For example, optogenetic ACR2 stimulation (onset, 100 ms; duration, 50 ms; intensity, 1.3 mV/mm^2^) was able to significantly (*P <* 0.01) shorten baseline APD_80_ values (414 ± 39 ms, *n =* 9) in the LQTS–hiPSC-CMs to 304 ± 36 ms (Figure 4F). Comparing APD_80_ values in the untreated and optogenetically treated ACR2-expressing LQTS–hiPSC-CMs with healthy control hiPSC-CMs (283 ± 9 ms, *n =* 10, Supplemental Figure 5), we noted that, while untreated LQTS–hiPSC-CMs displayed significantly (*P <* 0.05) longer values than healthy control cells, optogenetic treatment was able to fully correct APD values to levels that were not statistically different than control CMs.

### Optical monitoring of AP properties during optogenetic stimulation.

Since patch-clamp allows only short-term and low-throughput studies and may interfere with intracellular Cl^–^ concentration, we repeated some of the key experiments with a noninvasive optical method for monitoring AP properties. To this end, the line-scan model of a confocal microscope was used to derive optical APs from ChR2- or ACR2-expressing hiPSC-CMs that were loaded with the voltage-sensitive dye, FluoVolt (Supplemental Figure 6).

To prolong the optical APD, we evaluated ChR2 light activation in hiPSC-CMs using a prolonged (80–400 ms) pulsed-stimulation protocol (repeated cycles of 15 ms on/25 ms off), initiated 60 ms after AP onset. These illumination protocols significantly prolonged APD_70_ values in both healthy control hiPSC-CMs (Supplemental Figure 6, A and B) and SQTS–hiPSC-CMs (Supplemental Figure 6, C and D), with the degree of optical APD_70_ prolongation correlating with illumination duration (Supplemental Figure 6, B and D).

To shorten the optical APD, we exposed ACR2-expressing healthy hiPSC-CM (Supplemental Figure 6, E and F) and LQTS–hiPSC-CM (Supplemental Figure 6F) to light-activations (duration, 50ms), initiated 50–200 ms after AP onset. This led, in both cell types, to significant APD_70_ shortenings (Supplemental Figure 6, E and F), with earlier light applications resulting in greater APD_70_ shortening (Supplemental Figure 6G). Finally, early phase 2 light activation of ChR2-expressing LQTS–hiPSC-CMs also shortened APD_70_, but the degree of shortening was much smaller than that achieved by ACR2 (*P <* 0.01, Supplemental Figure 6G).

### Continuous illumination clamps the V_m_ and inhibits excitability.

**Figure 5:**
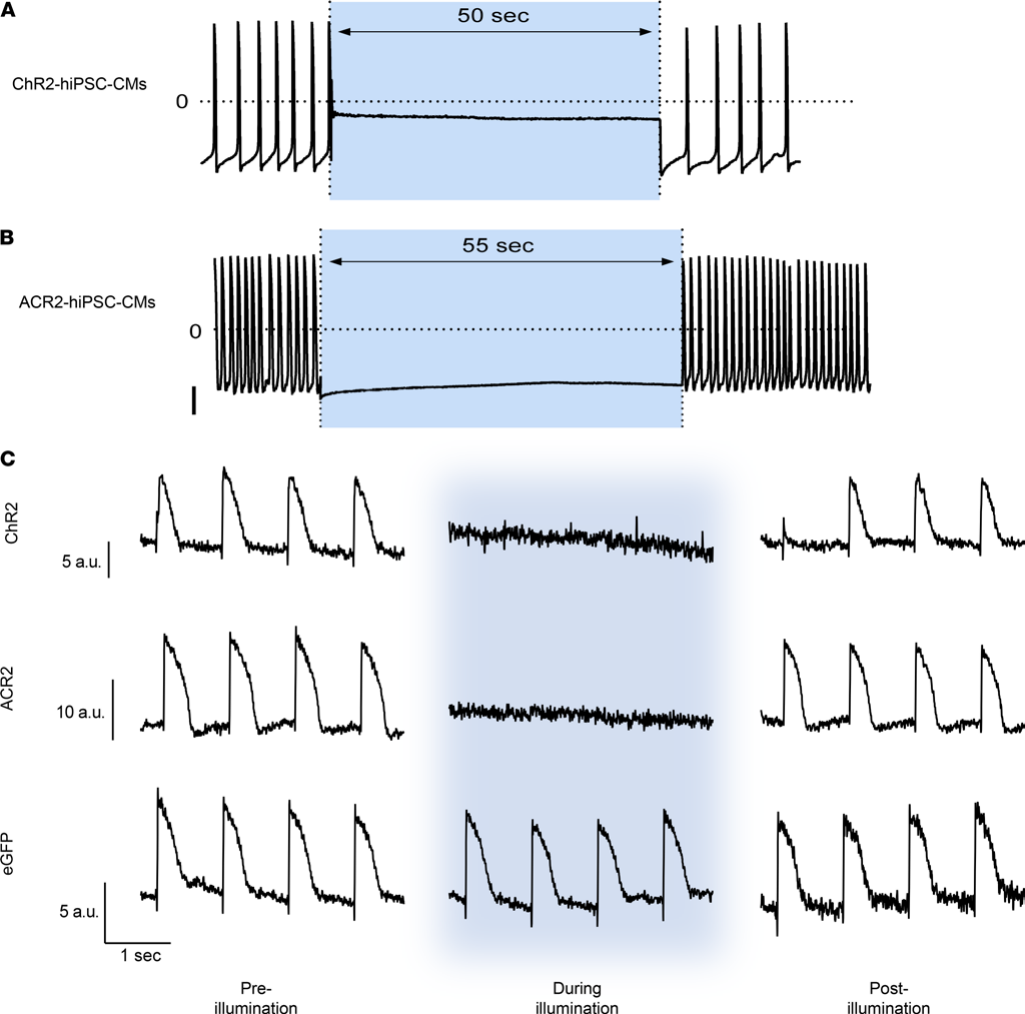
Optogenetic protocols to suppress cardiomyocyte excitability. (**A** and **B**) Whole-cell patch-clamp recordings from ChR2-expressing (**A**) or ACR2-expressing (**B**) hiPSC-CMs. Notice how continuous prolonged 1.3 mW/mm^2^ blue light illumination clamps membrane potential to either a depolarized (in the case of ChR2; **A**) or hyperpolarized (ACR2; **B**) potential and suppresses spontaneous AP generation. Scale bar: 40 mV. (**C**) Representative optical AP recording (using voltage-dye imaging), acquired during continuous electrical field stimulation (1 Hz) of hiPSC-CMs expressing either ChR2 (representing 7 experiments, top panel), ACR2 (representing 19 experiments, middle panel), or eGFP (representing 17 experiments, bottom panel). Note that prolonged illumination with 1.3 mW/mm^2^ blue light completely suppressed AP development in ChR2-expressing (top panel) and ACR2-expressing (middle panel) hiPSC-CMs. The same illumination protocol, however, did not affect control eGFP-expressing hiPSC-CMs (bottom panel). Illumination timing is represented in all tracings by the blue background.

Given the robust depolarizing and hyperpolarization effects achieved by transient opsin activation, we next tested the hypothesis that long-term illumination could be used to completely inhibit excitability by continuously clamping the CM’s V_m_. To this end, whole-cell patch-clamp studies were used to record APs from hiPSC-CMs, transduced to express either ChR2 (Figure 5A) or ACR2 (Figure 5B). As shown in Figure 5A, prolonged light-induced ChR2 activation clamped V_m_ to a relatively depolarized value and completely eliminated AP generation during this period. Upon termination of light stimulation, the cells returned to spontaneously fire APs. Similarly, prolonged light-induced activation of ACR2-expressing hiPSC-CMs also clamped V_m_ but, in this case, to a hyperpolarized potential (Figure 5B). This also inhibited AP generation, with resumption of spontaneous activity following cessation of illumination (Figure 5B).

Finally, we performed similar studies in hiPSC-CMs that were loaded with voltage-sensitive dyes for optical AP monitoring (Figure 5C and Supplemental Movies 1 and 2). Importantly, while the ChR2 and ACR2 expressing hiPSC-CMs were successfully paced (field stimulation, 1 Hz) at baseline (darkness), their electrical activity was completely suppressed during illumination, despite continuous delivery of electrical stimuli (Figure 5C, top/middle panels). Complete suppression of excitability was noted in 6 of 7 ChR2-expressing hiPSC-CMs and in all 19 ACR2-expressing hiPSC-CMs studied. Upon cessation of illumination, there was resumption of electrical-pacing capture and generation of APs in all cells (Figure 5C, top/middle panels). In contrast, in control eGFP-expressing hiPSC-CMs (*n =* 17), illumination did not affect cell excitability with the continuous generation of APs (Figure 5C, bottom panel).

### Optogenetic-based tissue APD modulation.

**Figure 6:**
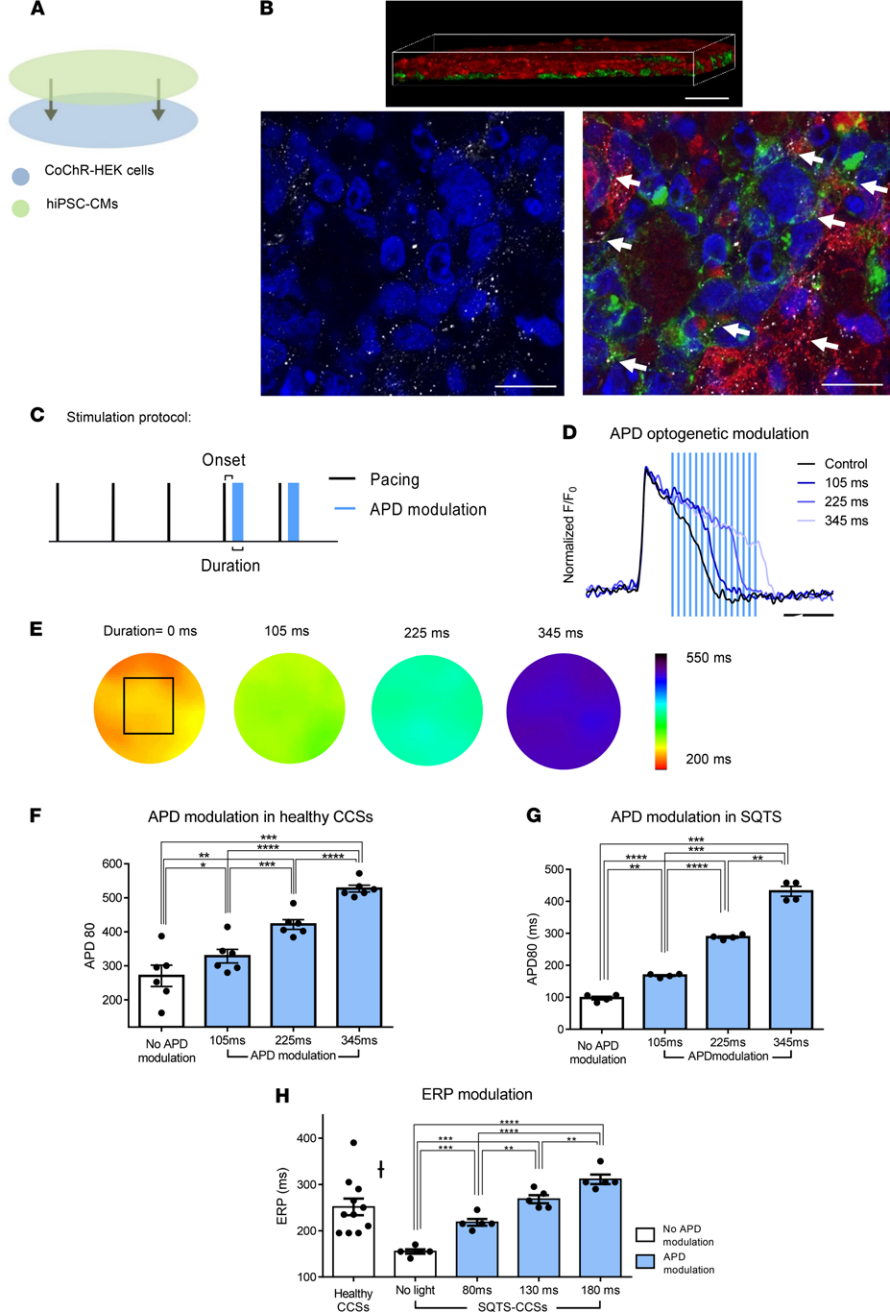
Optogenetics-based APD modulation at the tissue level. (**A**) Scheme describing the derivation of the in vitro coculture model. The hiPSC-derived cardiomyocyte cell sheets (hiPSC-CCSs) were seeded on top of a monolayer of CoChR-expressing HEK293 cells. (**B**) Confocal microscopy based 2D (bottom panels) and 3D reconstructed *z*-series (top panel) immunostainings of the cocultures. The hiPSC-CMs are identified as α-actinin^+^ cells (red) and engineered HEK293 cells by their eGFP expression (green). Gap junctions are indicated by the positive connexin 43 punctuate immunosignal (white) and indicated by arrows. Nuclei are counterstained with DAPI (blue). Scale bars: 50 mm (upper panel), 20 mm (lower panels). (**C**) Optogenetic-based APD modulation protocol. Both pacing (short flash [10 ms], black line) and APD (prolonged pulsed stimulus [100 ms], light-blue) modulation stimuli were achieved through diffuse light exposure of the culture. (**D**) Representative optical APs recordings (from 6 experiments) at baseline (black tracing) and during applications of the optogenetic APD modulating stimuli at variable durations (blue tracings). Note the correlation between the optical stimulus duration and the resulting APD prolongation. Scale bar: 200 ms. (**E**) Optical mapping–derived color-coded APD_80_ maps acquired at baseline (darkness, left) and during applications of the APD modulating signals (105, 225, and 345 ms). (**F**) Summary of changes in APD_80_ values at baseline (darkness) and following applications of the optogenetic stimuli in healthy control hiPSC–CCSs (*n =* 6; *P < 0.05, **P < 0.01, ***P < 0.001, ****P < 0.0001 using 1-way ANOVA for repeated measurements, followed by Tukey post hoc test). (**G** and **H**) Optogenetic-based modulations of APD_80_ (*n =* 4, **G**) and ERP (*n =* 5, **H**) values in the CoChR-SQTS–hiPSC-CCSs cocultures. Shown are baseline values and the effects of optogenetic stimuli of different durations (*P < 0.05, **P < 0.01, ***P < 0.001, ****P < 0.0001 using 1-way ANOVA for repeated measurements, followed by Tukey post hoc test). *P <* 0.01 using unpaired Student’s *t* test when comparing ERP values in SQTS versus healthy control hiPSC-CCS.

We next aimed to optogenetically control AP properties also at the tissue level. To this end, we utilized a recently described large-scale (~1 cm) circular-shaped hiPSC-derived CM cell sheet (hiPSC-CCS) model (34) and cocultured the layer of CMs on top of a monolayer of HEK293 cells, engineered to express the potent channelrhodopsin variant, CoChR (35) (Figure 6, A and B). Since HEK293 cells can generate gap junctions with neighboring CMs (11, 15, 36, 37), as manifested here by the high-resolution positive connexin 43 punctuate immunostaining (Figure 6B, bottom panels), CoChR light-activation in the engineered cells is expected to modulate AP properties of the coupled CMs through electrotonic interactions. We chose to use CoChR in these tissue coculture experiments rather than the original ChR2 used in the isolated CM experiments, since it displays greater light sensitivity and produces much larger currents when expressed in HEK cells (Supplemental Figure 7).

To monitor the electrical activity of the CoChR-HEK293/hiPSC-CCS cocultures, they were loaded with voltage-sensitive dyes and evaluated with a high-resolution optical mapping system (34). To test various optogenetic protocols, we utilized a digital micromirror device (DMD), allowing the generation of complex spatiotemporal illumination patterns. The protocol used to test optogenetic-based APD modulation included delivery of 4 consecutive diffuse optogenetic pacing stimuli (10 ms–long flashes), with the last pacing stimulus immediately followed (onset, 120 ms) by the APD-prolonging optogenetic signal (Figure 6C). As exemplified in the resulting optical APs traces (Figure 6D) and APD_80_ map (Figure 6E), light-induced CoChR activation significantly prolonged the tissue’s APD, with the degree of APD prolongation correlating with optogenetic signal duration (Figure 6, D–F). Consequentially, average APD_80_ values in the CoChR–hiPSC-CCSs increased significantly from a baseline value of 270 ± 31 ms to 328 ± 20 ms, 422 ± 14 ms, and 527 ± 10 ms following illumination durations of 105, 225, or 345 ms, respectively (Figure 6F).

We next evaluated the ability to optogenetically correct abnormal tissue AP properties by coculturing CoChR-HEK293 cells with patient-specific SQTS–hiPSC-CCSs. As expected, APD_80_ values in the SQTS–hiPSC-CCSs were extremely short (98 ± 6 ms, Figure 6G, *n =* 4) in comparison with healthy control tissues (270 ± 31 ms, Figure 6F, *n =* 6). Using the above-mentioned optogenetic APD-modulating protocol (Figure 6C), we could diffusely prolong and normalize APD_80_ values in the SQTS–hiPSC-CCSs (Figure 6G). Thus, application of APD-modulating stimuli — with illumination durations of 105, 225, and 345 ms — significantly prolonged the pathological APD_80_ values in the SQTS–hiPSC-CCS to 168 ± 3 ms, 288 ± 4 ms, and 431 ± 16 ms, respectively (Figure 6G).

Since a short refractory period is a key mechanism supporting reentrant arrhythmias in SQTS, we also evaluated the effects of optogenetic APD-modulating protocols on the tissue’s effective refractory period (ERP, Supplemental Figure 8). The SQTS–hiPSC-CMs exhibited shortened ERP values (170 ± 6 ms, *n =* 5, Figure 6H) compared with healthy control tissues (251 ± 18 ms, *n =* 11, Figure 6H, *P <* 0.01). Using the CoChR-optogenetic APD-modulating signal, the abbreviated ERP in the SQTS–hiPSC-CCSs could be significantly prolonged to 233 ± 7 ms, 283 ± 9 ms, and 326 ± 10 ms using illumination durations of 80, 130, and 180 ms, respectively (Figure 6H, *n =* 5).

### Dynamic optogenetic APD modulation can prevent reentrant arrhythmias.

**Figure 7:**
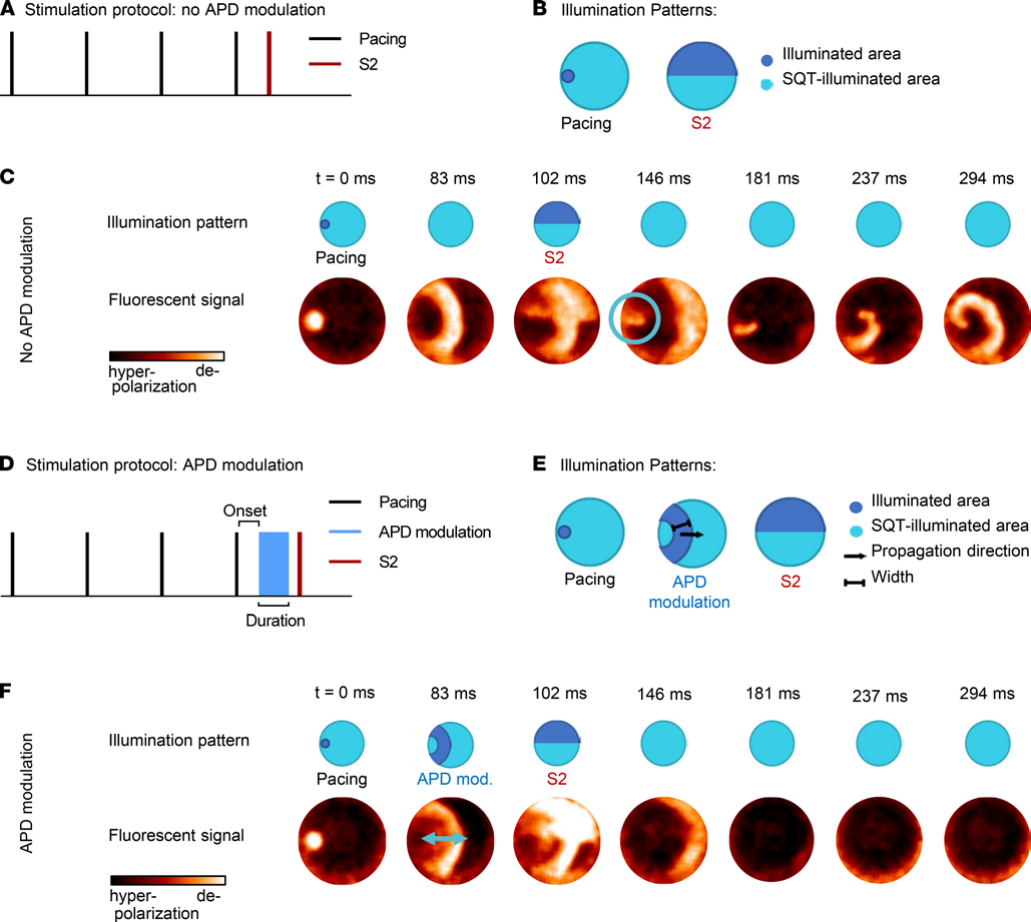
Dynamic optogenetic-based APD modulation for prevention of reentrant arrhythmias in the SQTS–hiPSC-CCS model. (**A**–**C**) Optogenetic cross-field protocol to induce spiral waves in the SQTS–hiPSC-CCSs. (**A** and **B**) Schemes describing the cross-field optogenetic stimulation protocols in both time (**A**) and space (**B**). The coculture is optogenetically paced using a point stimulation (S1) from the left side of the culture. When the S1-induced wavefront reaches the center of the tissue, a perpendicular wavefront is delivered by a broad S2 optogenetic-based stimulation wave originating from the top half of the culture (S2). (**C**) Sequential fluorescence images taken from the dynamic optical mapping display depicting the process of spiral wave induction. At t = 0 ms, a point optogenetic pacing stimuli (S1) induces a propagation wave traveling from left to right (t = 83 ms). When the propagation wave reaches the center of the culture, a broad optogenetic premature stimulation (S2) produced a new wavefront traveling perpendicular to the initial wave (t = 102 ms). This new wavefront is able preexcite already excitable tissue proximal to the traveling S1 wave (146 ms, marked in a blue circle) and initiate a sustained spiral wave (181–294 ms). (**D**–**F**) Optogenetic APD modulation prevents spiral wave induction. (**D** and **E**) Schemes depicting the optogenetic cross-field stimulation and dynamic APD modulation protocols in both time (**D**) and space (**E**). Following application of S1, an APD-modulating illumination pattern was delivered, which was designed to be identical to the shape of the S1-induced propagating activation wavefront and to follow this wavefront with the same CV and a fixed delay of 40 ms. To complete the cross-field stimulation, a perpendicular wavefront was then induced by S2 as described above. (**F**) Sequential fluorescence images taken from the dynamic optical-mapping display depicting the prevention of the cross-field–induced spiral wave generation by the APD-modulating signal. The prolongation of the tissue wavelength by the APD-modulating signal is marked with a double-headed blue arrow. This resulted in the prevention of the development of reentrant activity following the premature S2 excitation wavefront.

Based on the ability to prolong APD and ERP at the tissue level, we next aimed to develop a dynamic APD-modulating optogenetic strategy that could prevent reentrant arrhythmias. To this end, we initially developed a reproducible arrhythmia model in our SQTS–hiPSC-CCS cocultures using an optogenetic modification (38) of the well-established electrical cross-field stimulation strategy (39). This optogenetic protocol, which is schematically outlined in both time and space in Figure 7, A and B, allowed the generation of reentrant activity in SQTS–hiPSC-CCS cocultures in a robust, reproducible, and controlled manner.

An example of how a reentrant activity is induced by the aforementioned approach can be appreciated by the optical mapping results, presented as a dynamic display (Supplemental Movie 3) or as sequential fluorescence images (Figure 7C). Note that the SQTS–hiPSC-CCS/CoChR-HEK293 coculture is initially optogenetically paced using a point stimulation (S1) originating from the left side of the culture. When the S1-derived wavefront reaches the center, a broad perpendicular wavefront is initiated from the top of the culture by an S2 optogenetic stimulus. The S2 propagating wave then impinges on the tale of the S1 activation wave, initiating spiral wave reentry. Using this approach, we were able to generate sustained reentry, repeatedly, in all SQTS–hiPSC-CCSs cocultures (*n =* 15 from 5 independent experiments; Figure 7C and Supplemental Movie 3).

We next tested the hypothesis that a dynamic APD-modulating optogenetic-based protocol can be formulated that can prevent reentry in the SQTS–hiPSC-CCS model. To this end, we utilized the DMD apparatus to deliver a dynamic optogenetic stimulation protocol, which was patterned to faithfully follow the S1 propagating wavefront in both time (delivered at each pixel with a 40 ms delay after AP onset) and space, propagating in the same direction and with the same conduction velocity (CV) as the activation wavefront (Figure 7, D–F). Application of this dynamic illumination protocol increased both the APD and the resulting tissue WL (a product of CV and APD) of the propagating wave, as depicted in the respective fluorescent maps (Figure 7F, double-headed arrow).

To evaluate whether the aforementioned intervention can prevent arrhythmia initiation in the SQTS–hiPSC-CCSs, we repeated the same cross-field stimulation protocol but added the dynamic APD-modulating illumination (Figure 7, D–F) aimed at prolonging the shortened APD and WL associated with the SQTS that allows the development of reentrant arrhythmias. As depicted in the resulting optical-mapping dynamic display (Supplemental Movie 3) and sequential fluorescence images (Figure 7F), the APD-prolonging protocol was able to prevent initiation of spiral wave reentry by S2 activation. This antiarrhythmic effect resulted from the increase in the size of the refractory tissue at the tail of the S1 propagating wave, preventing reexcitation of this proximal area by the S2-derived activation wave and, consequentially, its ability to induce reentry.

**Figure 8:**
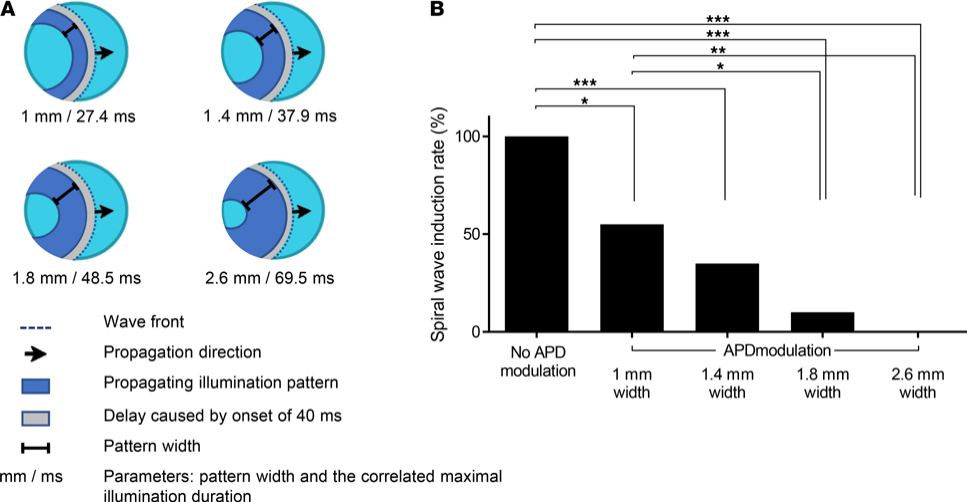
Adjustable optogenetic prevention of reentrant arrhythmias in the SQTS–hiPSC-CCS model. (**A** and **B**) Effects of changing the properties of the APD-modulation illumination signal on its antiarrhythmic capability. (**A**) Schemes highlighting the different optogenetic APD modulating patterns used and the resulting maximal APD prolongation (in ms) and degree of wavelength prolongation (in mm) of the propagative wavefront in the SQTS tissue model for each intervention. (**B**) Summary of the spiral wave induction rate using each of the different APD modulation protocols. The protocol with no APD modulation served as control (*n =* 20 in 6 independent experiments). *P < 0.05, **P < 0.01, ***P < 0.001 using Cochran’s Q test for repeated measurements of binary data, followed by Dunn post hoc test).

We next tested the effects of altering the parameters of the dynamic illumination protocol on its antiarrhythmic capabilities. Specifically, we compared the arrhythmogenic outcome of the cross-field stimulation protocol that was not supplemented by an APD-modulating illumination protocol (control group) with 4 protocols that also included dynamic APD-prolonging interventions of various illumination durations (Figure 8A). Both APD prolongation (in ms) and the associated WL prolongation (in mm) resulting from the different optogenetic interventions are schematically highlighted in Figure 8A. Our results revealed development of spiral waves following cross-field stimulation in all control (without APD modulation, *n =* 20) experiments.

Application of the dynamic APD-modulating protocols significantly reduced the incidence of arrhythmia induction (Figure 8B). Interestingly, there was a high correlation between the degree of WL increase and the intervention’s antiarrhythmic potential (Figure 8B). For example, while an illumination protocol designed to increase the WL by 1 mm was associated with a 55% incidence (11 of 20) of spiral wave induction, increasing the tissue WL to 2.6 mm completely inhibited (0 of 20) arrhythmia inducibility (Figure 8B). Interestingly, the degree of WL prolongation required to completely prevent spiral wave induction did not have to be extremely long and did not even reach the level of the WL in healthy control tissue (Supplemental Figure 9)

## Discussion

Disruption of AP morphology in a variety of inherited or acquired conditions, such as in the LQTS and SQTS, can lead to life-threatening arrhythmias. In the current study, we describe the use of 2 ground-breaking technologies, patient/disease-specific hiPSCs and optogenetics, to develop functional, optogenetic-based strategies to shape AP morphology in human CMs and to correct the abnormal AP properties and increased arrhythmogenic risk associated with the LQTS and SQTS.

We initially focused on the light-sensitive cationic channel, ChR2 (29). Similar to its role in neuroscience, ChR2 — in cardiac optogenetics — has primarily been used to augment or inhibit AP generation for optogenetic-based pacing (13, 14), resynchronization (14, 15), and defibrillation (16–20) approaches. Here, we activated ChR2 during the AP with the aim of modulating AP properties. Surprisingly, our results revealed that ChR2 activation can be used bidirectionally, depending on illumination timing, producing either depolarizing currents (leading to APD prolongation), or hyperpolarizing currents (APD shortening). These opposing effects result from the relationship between the CM’s V_m_ at the time of optogenetic stimulation relative to ChR2-E_Rev_. Optical stimulations applied while V_m_ is more positive than ChR2-E_Rev_ (early phase 2 of the AP) result in hyperpolarizing currents, while depolarizing photocurrents are produced if V_m_ is more negative than ChR2-E_Rev_ (phases 3 and 4).

The ability to prolong APD in the ChR2-expressing hiPSC-CMs was highly robust and predictable, with the resulting APD correlating with the timing of the end of the optogenetic stimulus. This allowed to tailor the specific optical stimulation protocol to achieve any desired APD prolongation and, consequentially, to correct the abnormally shortened APD in the SQTS–hiPSC-CMs to levels comparable with healthy control cells.

As mentioned, APD could also be shortened in the ChR2-expressing hiPSC-CMs by optical stimuli delivered early during the AP. This effect, however, was relatively limited and APD could not be shortened beyond a minimal value, even when increasing light intensity or stimulation duration. This ceiling effect probably stems from the relatively short time window in which V_m_ resides above ChR2-E_Rev_. Consequentially, using this approach, we could shorten the abnormally prolonged APD in LQTS–hiPSC-CMs but not to levels seen in healthy control hiPSC-CMs. Nevertheless, this optogenetic hyperpolarizing effect was still of therapeutic value, as it could suppress arrhythmic activity (EAD formation) in the LQTS–hiPSC-CMs. Importantly, optogenetic stimulation did not affect other components of the AP, such as AP’s amplitude, maximum rise slope, or the maximal diastolic potential — indicating that the major ionic currents responsible for phase 0 (Na^+^ channels) and late repolarization and the resting V_m_ (K^+^ currents) were not affected.

To achieve more significant APD shortening in healthy/diseased hiPSC-CMs, we utilized the anion-selective opsin ACR2. ACR2 is part of the ACRs family that displays strict anion selectivity and high unitary conductance, resulting in inhibition of neuronal activity at much lower light intensities than previously used opsins (31). More recently, ACR2 expression and light activation in neonatal rat CMs was shown to produce hyperpolarizing photocurrents, allowing for significant AP modulation and silencing (23). Here, we demonstrate the robust effects of ACR2 light activation in shortening APD in healthy and diseased hiPSC-CMs. In contrast to the ceiling effect and limited APD shortening achieved by early ChR2 activation, ACR2-induced APD shortening effects were robust, unlimited, and could be modulated by parameters such as light intensity and optical stimulus onset and duration. Consequentially, the degree of APD shortening could be tailored to achieve any desired value — allowing for the normalization of APD duration in the LQTS–hiPSC-CMs to levels that were not different from healthy control hiPSC-CMs.

In addition to the APD-modulating effects achieved by optogenetic stimuli lasting up to hundreds of milliseconds, we could also completely inhibit cellular automaticity/excitability by ChR2 or ACR2 activation using stimuli lasting several seconds. Whereas, mechanistically, continuous ChR2 activation inhibited AP generation by clamping V_m_ to a relatively depolarized potential, leading to inactivation of Na^+^ channels, ACR2 activation clamps V_m_ to a hyperpolarized potential, preventing the required depolarization for AP generation. These results may have important implications for better mechanistic understanding and for adding new optogenetic tools (use of ACR2) for optogenetic-based therapeutic strategies for tachyarrhythmias: for suppression of automaticity, generation of functional conduction blocks, and optogenetic defibrillation (12, 40).

Some of the important technical advancements introduced in this study were the ability to use pulsed optogenetic stimulation as an alternative to continuous illumination and the use of an all-optical system approach as an alternative to patch-clamp recordings. This allowed monitoring and accurate reconstruction of optical APs of the studied cells, validating the optogenetic stimulation effects also in unperturbed intact cells. In addition, the pulsed stimulation alternative, which can be reduced to very short duty cycles (1%), can reduce the transmitted light energy. The latter property, in addition to allowing all-optical recording without illumination artifacts, may be important for future in vivo studies.

Most importantly, we also demonstrated the ability to optogenetically modulate AP properties at the tissue level. To this end, we utilized a modification of a recently described hiPSC-CCS model (34), in which hiPSC-CMs were cocultured with opsin-carrying cells (CoChR-HEK293 cells). We chose to use CoChR (a potentially new ChR variant) in these studies because we hypothesized that a more potent light-sensitive channel (producing larger photocurrents at lower illumination intensity) will be required in the tissue coculture model in comparison with the single-cell studies. The need for expression of a more potent opsin in the engineered HEK cells stems both from the potential illumination attenuation as light passes through the CM layers until it reaches the HEK-cell layer at the bottom of the coculture and the need to generate large enough electrotonic currents to affect adjacent CMs through electrotonic interactions.

Initially, we showed the ability to prolong APD diffusely throughout the tissue in both healthy control and SQTS–hiPSC-CCSs. The degree of APD prolongation in both models highly correlated with illumination duration, allowing to tailor APD to any desired value and to completely normalize the abbreviated APD associated with the SQTS.

After confirming the ability to diffusely affect the tissue’s APD, our more ambitious goal was to develop illumination protocols that could alter APD in a regional manner and to change dynamically in both space and time. To achieve this goal, we utilized the DMD apparatus to design and pattern dynamic optogenetic stimulation protocols that could closely follow and modulate the electrical properties of the propagating activation wavefront. To test the hypothesis that such interventions could be antiarrhythmic, we also established a reproducible reentrant arrhythmia model (spiral waves) in the SQTS–hiPSC-CCS cocultures using an optogenetic variation (38) of the well-established electrical cross-field stimulation strategy (39).

We next optimized the dynamic optogenetic APD-modulating stimulation protocol to closely follow the propagating activation wavefront, aiming to prolong the shortened APD and tissue WL associated with the SQTS. This resulted in prolongation of the tissue’s APD and WL (estimated as the product of CV and APD) — a property that is antiarrhythmogenic in nature (30). The result of this spatially patterned APD-modulating illumination protocol was an increase in the size of the area behind the S1 propagating wavefront that was still refractory, thereby decreasing the ability of the S2-induced perpendicular wavefront to propagate proximal to the traveling wavefront and to induce reentry. Importantly, we found a direct correlation between the magnitude of the prolonging effect on the tissue’s WL and the degree of the antiarrhythmic effect achieved. Interestingly, the illumination pattern that already led to maximum efficiency (complete prevention of arrhythmia induction) did not require huge APD and WL (2.6 mm) prolonging effects. In fact, the resulting APD and WL values in the SQTS tissues that were sufficient to completely prevent reentry were even shorter than the corresponding values of unperturbed healthy tissue.

In summary, our results provide a robust, functional, and reversible approach to modulate AP properties in human CMs by optogenetic tools at both the cellular and tissue levels. By utilizing different opsins with unique biophysical properties and applying variable light stimulation patterns, we could tailor the procedure to shape AP morphology, to derive any desired APD, to prevent arrhythmogenicity at both the cellular (EADs and triggered-activity in the LQTS) and tissue (reentry in the SQTS) levels, and even to completely silence electrical activity. Future applications, however, would require moving from the single-cell and tissue levels to the whole-heart (in vivo) level. Expanding this approach will allow researchers to model arrhythmogenic conditions associated with dispersion of repolarization and to develop novel anti-arrhythmic strategies.

## Methods

Supplemental Methods are available online with this article.

### Propagation of hiPSCs and CM differentiation.

Previously established healthy control (25, 26), LQTS (25), and SQTS (30) hiPSC lines were used. Undifferentiated hiPSCs colonies were propagated using mTeSR-1 medium. For CM differentiation, a modification of a monolayer-directed differentiation system was used (27, 28). In brief, the differentiation RPMI/B27 medium containing RPMI-1640, 2%-B27 supplement minus insulin (Invitrogen), 1% penicillin/streptomycin, and 6 mM CHIR99021 (Stemgent) was used for 2 days. Medium was changed to RPMI/B27 (without CHIR) supplemented with 2 μM Wnt-C59 (Selleckchem) on days 3–4. Beating monolayers (20–60 days) were enzymatically dissociated into small clusters or single CMs using TrypLE (Thermo Fisher Scientific) and plated on matrigel-coated MatTek plates for the different studies.

### Lentiviral transduction of the ChR2 and the ACR2 transgenes.

The pLV-CAG-*ChR2*-*GFP*, pLV-CAG-*ACR2-YFP*, and pTK113-*GFP* plasmids were used for virus production in 80% confluent HEK293T cells. The relevant plasmid was cotransfected with the packaging cassette NRF and the VSVG plasmid using PolyJet reagent (SignaGen Laboratories). DNA (8 μg) was used per 10 cm dish, where the transgene of interest, NRF, and VSVG plasmids were mixed in a 3:2:1 ratio. Fresh virus-containing media were collected at 48 and 72 hours and used for 2 rounds of infections of dissociated hiPSC-CMs.

### Whole-cell patch-clamp recordings.

Recordings were conducted with MultiClamp 700B and Digidata 1440A (Axon Instruments). Dissociated hiPSC-CMs were perfused with bath solution containing (in mM): NaCl 140, KCl 3, HEPES 10, glucose 10, MgCl_2_ 2, and CaCl_2_ 2 (pH 7.4, adjusted with NaOH) and maintained at 35°C (Warner Instruments). The pipette solutions used are provided in Supplemental Table 1. The hiPSC-CMs were paced at 1 Hz. AP recordings (current-clamp) were performed at baseline (darkness) and during predesigned illumination (470 nm monochromatic light at 1.3 mW/mm^2^) protocols. APD_80_ was calculated using the Clampfit 10.7 software (Molecular Devices).

To measure ChR2 or ACR2 photocurrents, voltage clamp and AP clamp experiments were conducted. Photocurrents were calculated by subtracting the measurements during darkness from those under blue light illumination. Current-voltage (I-V) curves describing peak- and steady-state photocurrents were constructed for V_m_ values ranging from –80 mV to 60 mV at 10 mV increments. For AP clamp studies, prerecorded AP waveforms served as voltage commands.

### Confocal optical monitoring of hiPSC-CMs.

Cells were loaded with the voltage-sensitive dye FluoVolt (Invitrogen, 30 minutes, 37°C). Medium was replaced by extracellular Tyrode’s solution containing (in mM): NaCl 140, KCl 3, HEPES 10, glucose 10, MgCl_2_ 2, and CaCl_2_ 2 (pH 7.4, adjusted with NaOH). The line-scan model of the confocal microscope (LSM710, Zeiss) was used to acquire optical APs by monitoring the fluorescence intensity of FluoVolt (Excitation: 543 nm; emission measured through a BP620/52 filter, Chroma Technology). Experiments were performed after 5 seconds of steady-state 1 Hz (healthy control and SQTS hiPSC-CMs) or 0.5 Hz (LQTS–hiPSC-CMs) electrical field-stimulation. APD modulating pulsed optogenetic stimulations were applied at 1.3 mW/mm^2^. Custom-made Matlab software was used to eliminate light artifacts and to fill the gaps in the optical traces by Matlab “fill-gaps” algorithm. APD_70_ values were compared at baseline (darkness) and during optical stimulation.

### Optogenetics illumination.

Optogenetics illumination for the single-cell experiments was performed using a 470 nm fiber-coupled LED connected to high-power LED-driver (Thorlabs). A programmable stimulus-generator (STG-1004, multichannels systems) was used to control illumination timing, duration, and intensity and to couple its delivery to the electrical stimulus used for AP generation. The onset of optogenetic stimulation was defined as the time interval between the electrical pacing stimulus and beginning of optogenetic stimulation. For experiments at the tissue level (hiPSC-CCSs), illumination patterns were generated by a digital micro mirror device (DMD, Polygon-400, Mightex systems) controlled by PolyScan software. Illumination was pulsed (9 ms on/15 ms off).

### Establishing the engineered HEK293 cells.

CoChR-expressing HEK293 cells were established using lentiviral transduction of the CoChR-GFP transgene. Cells were sorted based on eGFP fluorescence using FACSAria (BD Biosciences) and further purified using a 10-day G418 antibiotic (800 μg/mL) selection process.

### Establishing the CoChR-HEK293/hiPSC-CCSs coculture models.

In total, 180,000 CoChR-HEK293 cells were seeded in the 10 mm inner well of Matrigel-coated MatTet plate (P35G-1.5–10-C, MatTek) as a monolayer. Twenty-four hours later, seeded cells were treated with 24 μM Mitomycin C (MilliporeSigma) for 1 hour to prevent cell proliferation. At day 2, 1 × 10^6^ hiPSC-CMs (either control or SQTS) were seeded on top of the CoChR-HEK monolayer to generate the cocultures. The culture medium was supplemented with 5 μM Blebbistatin (B0560-5MG, MilliporeSigma) to prevent vigorous contraction.

### Optical mapping of the hiPSC-CCSs cocultures.

Cocultures were loaded with the voltage-sensitive dye Di4-ANBDQBS (22.5 μg/mL, acquired from Leslie Loew, University of Connecticut, Farmington, Connecticut, USA) for 15 minute at room temperature. Optical mapping was performed using an Electron Multiplying CCD (EMCCD) Camera (Evolve-512 Delta, Photometrics) and a macroscope (Olympus MVX10). The X-Cite Turbo LED system served as light source. Excitation filter for Di4-ANBDQBS was Chroma ET620/60× and emission filter was Chroma ET665lp. Micro Manager software was used for acquisition, and the OMProCCD software, a custom-made IDL based software (provided by Bum-Rak Choi, Brown University, Providence, Rhode Island, USA) served for analysis.

To analyze the data, light artifacts from the optogenetic stimulation were eliminated and replaced by a threshold-based custom-made Matlab algorithm. Optical signals at each pixel were then analyzed to measure the local activation time (timing of the maximal dF/dt value) and APD_80_ (the time difference between maximal dF/dt and 80% decay from peak to baseline). These values were used to generate detailed activation and APD_80_ maps.

### Immunostaining.

Cultures were fixed with 4% paraformaldehyde (Bio-Lab). Blocking was performed by 5% horse serum (Thermo Fisher Scientific, 1 hour). An incubation period with primary antibodies was carried out overnight at 4°C. The primary antibodies were used for staining of connexin-43 (1:100; rabbit; Santa Cruz Biotechnology, sc-9059) and α-actinin (1:100; mouse; Sigma-Aldrich, A7811). Samples were washed (×3) with PBS and incubated (1 hour) with 1:150 diluted secondary antibodies: Cy3 donkey anti–mouse IgG (715-165-151, Jackson ImmunoResearch) and Cy5 donkey anti–rabbit IgG (711-175-152, Jackson ImmunoResearch). Antibodies were diluted in PBS with 3% horse serum and 0.1% Triton. Nuclei were stained with DAPI (1:500, MilliporeSigma, D9564). Zeiss LSM-710 laser-scanning confocal microscope (Zeiss) was used for imaging.

### Statistics.

Statistical analysis was performed using GraphPad Prism software, except for the Cochran’s Q test for binary data, which was performed on SPSS. Data were presented as mean ± SEM. Differences between different groups were compared using unpaired 2-tailed Student’s *t* test. For studies comparing measurements from the same cells at baseline (darkness) and following optogenetic illuminations, we used the paired 2-tailed Student’s *t* test. For studies involving multiple comparisons of 1 or 2 independent variables, either 1-way or 2-way ANOVA was performed, respectively, followed by either post hoc Tukey or Dunnett’s (for multiple comparisons with the same control group) tests. To evaluate a potential correlation between the optogenetic stimulation parameters and the resulting changes in APD values, the Pearson’s correlation coefficient was calculated, and a linear regression model was used. For multiple comparisons of paired binary data (i.e., spiral wave induction rate following different illumination protocols), Cochran’s Q test was performed followed by a post hoc Dunn test. A value of *P <* 0.05 was considered statistically significant.

## Author contributions

A. Gruber, OE, and LG designed the experiments. GA and A. Gepstein performed the hiPSC CM differentiation. IH performed the lentiviral transduction studies. A. Gruber, OE, AS, ML, SC, and NS performed the electrophysiological studies and analysis. A. Gruber, OE, and LG wrote the manuscript. LG supervised the work.

## Supplementary Material

Supplemental data

Supplemental Video 1

Supplemental Video 2

Supplemental Video 3

## Version 1 06/08/2021

Electronic publication

## References

[1] Schram G (2002). Differential distribution of cardiac ion channel expression as a basis for regional specialization in electrical function. Circ Res.

[2] Goldenberg I, Moss AJ (2008). Long QT syndrome. J Am Coll Cardiol.

[3] Gaita F (2003). Short QT Syndrome. Circulation.

[4] Sanguinetti MC, Tristani-Firouzi M (2006). hERG potassium channels and cardiac arrhythmia. Nature.

[5] Patel C (2010). Short QT syndrome: from bench to bedside. Circ Arrhythm Electrophysiol.

[6] Boyden ES (2005). Millisecond-timescale, genetically targeted optical control of neural activity. Nat Neurosci.

[7] Deisseroth K (2015). Optogenetics: 10 years of microbial opsins in neuroscience. Nat Neurosci.

[8] Zhang F (2011). The microbial opsin family of optogenetic tools. Cell.

[9] Arrenberg AB (2010). Optogenetic control of cardiac function. Science.

[10] Bruegmann T (2010). Optogenetic control of heart muscle in vitro and in vivo. Nat Methods.

[11] Jia Z (2011). Stimulating cardiac muscle by light: cardiac optogenetics by cell delivery. Circ Arrhythm Electrophysiol.

[12] Entcheva E, Kay MW (2021). Cardiac optogenetics: a decade of enlightenment. Nat Rev Cardiol.

[13] Vogt CC (2015). Systemic gene transfer enables optogenetic pacing of mouse hearts. Cardiovasc Res.

[14] Nussinovitch U, Gepstein L Optogenetics for in vivo cardiac pacing resynchronization therapies (2015). Nat Biotechnol.

[15] Nussinovitch U (2014). Modulation of cardiac tissue electrophysiological properties with light-sensitive proteins. Cardiovasc Res.

[16] Bruegmann T (2016). Optogenetic defibrillation terminates ventricular arrhythmia in mouse hearts and human simulations. J Clin Invest.

[17] Funken M (2019). Optogenetic hyperpolarization of cardiomyocytes terminates ventricular arrhythmia. Front Physiol.

[18] Crocini C (2016). Optogenetics design of mechanistically-based stimulation patterns for cardiac defibrillation. Sci Rep.

[19] Nyns EC (2017). Optogenetic termination of ventricular arrhythmias in the whole heart: towards biological cardiac rhythm management. Eur Heart J.

[20] Watanabe M (2017). Optogenetic manipulation of anatomical re-entry by light-guided generation of a reversible local conduction block. Cardiovasc Res.

[21] Karathanos TV (2014). Optogenetics-enabled dynamic modulation of action potential duration in atrial tissue: feasibility of a novel therapeutic approach. Europace.

[22] Quach B (2018). Light-activated dynamic clamp using iPSC-derived cardiomyocytes. Biophys J.

[23] Govorunova EG (2016). Anion channelrhodopsins for inhibitory cardiac optogenetics. Sci Rep.

[24] Park SA (2014). Optical mapping of optogenetically shaped cardiac action potentials. Sci Rep.

[25] Itzhaki I (2011). Modelling the long QT syndrome with induced pluripotent stem cells. Nature.

[26] Zwi-Dantsis L (2013). Derivation and cardiomyocyte differentiation of induced pluripotent stem cells from heart failure patients. Eur Heart J.

[27] Burridge PW (2014). Chemically defined generation of human cardiomyocytes. Nat Methods.

[28] Shinnawi R (2015). Monitoring human-induced pluripotent stem cell-derived cardiomyocytes with genetically encoded calcium and voltage fluorescent reporters. Stem Cell Reports.

[29] Nagel G (2003). Channelrhodopsin-2, a directly light-gated cation-selective membrane channel. Proc Natl Acad Sci U S A.

[30] Shinnawi R (2019). Modeling reentry in the short QT syndrome with human-induced pluripotent stem cell-derived cardiac cell sheets. J Am Coll Cardiol.

[31] Govorunova EG (2015). Neuroscience. Natural light-gated anion channels: a family of microbial rhodopsins for advanced optogenetics. Science.

[32] Caille JP (1981). Intracellular chloride activity in rabbit papillary muscle: effect of ouabain. Am J Physiol.

[33] Baumgarten CM, Fozzard HA (1981). Intracellular chloride activity in mammalian ventricular muscle. Am J Physiol.

[34] Shaheen N (2018). Human induced pluripotent stem cell-derived cardiac cell sheets expressing genetically encoded voltage indicator for pharmacological and arrhythmia studies. Stem Cell Reports.

[35] Klapoetke NC (2014). Independent optical excitation of distinct neural populations. Nat Methods.

[36] Nussinovitch U, Gepstein L (2015). Optogenetics for suppression of cardiac electrical activity in human and rat cardiomyocyte cultures. Neurophotonics.

[37] Yankelson L (2008). Cell therapy for modification of the myocardial electrophysiological substrate. Circulation.

[38] Feola I (2017). Localized optogenetic targeting of rotors in Atrial Cardiomyocyte Monolayers. Circ Arrhythm Electrophysiol.

[39] Frazier DW (1989). Stimulus-induced critical point. Mechanism for electrical initiation of reentry in normal canine myocardium. J Clin Invest.

[40] Gruber A (2018). Cardiac optogenetics: the next frontier. Europace.

