# Supplementary material for: Optogenetic modulation of cardiac action potential properties may prevent arrhythmogenesis in short and long QT syndromes
Source: JCI Insight. 2021 Jun 8;6(11):e147470. doi: 10.1172/jci.insight.147470 (PMC8262308; doi:10.1172/jci.insight.147470)
Supplement: Supplemental data [file jciinsight-6-147470-s198.pdf]

SUPPLEMENTARY MATERIAL

Supplementary Materials and Methods

Propagation of hiPSCs and cardiomyocyte differentiation

Previously established healthy-control[1], LQTS[1], and SQTS[2] hiPSC lines were used. Undifferentiated hiPSCs colonies were propagated using mTeSR-1 medium. For cardiomyocyte differentiation, a modification of a monolayer directed differentiation system was used[3,4]. In brief, the differentiation RPMI/B27 medium containing RPMI-1640, 2%-B27 supplement minus insulin (Life-Technologies), 1% penicillin/streptomycin, and 6mM-CHIR99021 (Stemgent) was used for 2d. Medium was changed to RPMI/B27 (without CHIR) supplemented with 2µM-Wnt-C59 (Selleckchem) on days 3–4. Beating monolayers (20–60d) were enzymatically dissociated into small-clusters or single-cardiomyocytes using TrypLE and plated on matrigel-coated MatTek plates for the different studies.

Lentiviral transduction of the ChR2 and the ACR2 transgenes

The pLV-CAG-*ChR2-GFP*, pLV-CAG-*ACR2-YFP*, and pTK113-*GFP* plasmids were used for virus production in 80% confluent HEK293T cells. The relevant plasmid was co-transfected with the packaging cassette NRF and the VSVG plasmid using PolyJet reagent (SignaGen-Laboratories). 8µg DNA was used per 10cm dish, where the transgene of interest, NRF, and VSVG plasmids were mixed in a 3:2:1 ratio. Fresh virus-containing media were collected at 48 and 72h and used for two rounds of infections of dissociated hiPSC-CMs.

Whole-cell patch-clamp recordings

Recordings were conducted with MultiClamp700B and Digidata1440A (Axon-Instruments). Dissociated hiPSC-CMs were perfused with bath-solution containing (in mM): NaCl-140, KCl-3, HEPES-10, glucose-10, MgCl2-2, and CaCl2-2 (pH-7.4, adjusted with NaOH) and maintained at 35°C (Warner Instruments). The pipette solutions used are provided in

Supplementary Table 1. The hiPSC-CMs were paced at 1Hz. AP recordings (current-clamp) were performed at baseline (darkness) and during pre-designed illumination (470nm monochromatic light at 1.3mW/mm^2) protocols. APD_{80} was calculated using the Clampfit-10.7 software (Molecular-Devices).

To measure ChR2 or ACR2 photocurrents, voltage- and AP- clamp experiments were conducted. Photocurrents were calculated by subtracting the measurements during darkness from those under blue-light illumination. Current-voltage (I-V) curves describing peak- and steady-state photocurrents were constructed for V_m values ranging from -80mV to 60mV at 10mV increments. For AP-clamp studies, pre-recorded AP waveforms served as voltage commands.

Confocal optical monitoring of hiPSC-CMs

Cells were loaded with the voltage-sensitive dye FluoVolt (Invitrogen, 30min, 37°C). Medium was replaced by extracellular Tyrode's solution containing (in mM): NaCl-140, KCl-3, HEPES-10, glucose-10, MgCl2-2, and CaCl2-2 (pH-7.4, adjusted with NaOH). The line-scan model of the confocal microscope (LSM710, Zeiss) was used to acquire optical APs by monitoring the fluorescent intensity of FluoVolt (Excitation: 543nm; emission measured through a BP620/52 filter, Chroma-Technology). Experiments were performed after 5s steady-state 1Hz (healthy-control and SQTS hiPSC-CMs) or 0.5Hz (LQTS-hiPSC-CMs) electrical field-stimulation. APD-modulating pulsed optogenetic stimulations were applied at 1.3mW/mm$^{2.}$ A custom-made Matlab software was used to eliminate light artifacts and filling the gaps in the optical traces by Matlab "fillgaps" algorithm. APD_{70} values were compared at baseline (darkness) and during optical stimulation.

Optogenetics illumination

Optogenetics illumination for the single-cell experiments was performed using a 470nm fiber-coupled LED connected to high-power LED-driver (Thorlabs). A programmable stimulus-

generator (STG-1004, multichannels systems) was used to control illumination timing, duration and intensity and to couple its delivery to the electrical stimulus used for AP generation. The onset of optogenetic stimulation was defined as the time-interval between the electrical pacing stimulus and beginning of optogenetic stimulation. For experiments at the tissue level (hiPSC-CCSs), illumination patterns were generated by a digital micro mirror device (DMD, Polygon-400, Mightex systems) controlled by PolyScan software. Illumination was pulsed (9ms on/15ms off).

Establishing the engineered HEK293 cells

CoChR-expressing HEK293 cells were established using lentiviral transduction of the CoChR-GFP transgene. Cells were sorted based on eGFP-fluorescense using FACSAria (BD-Biocsiences) and further purified using a 10-day G418 antibiotic (800μg/ml) selection process.

Establishing the CoChR-HEK293/hiPSC-CCSs co-culture models

180,000 CoChR-HEK293 cells were seeded in the 10mm inner-well of Matrigel-coated MatTet plate (P35G-1.5-10-C, MatTek) as a monolayer. 24h later, seeded cells were treated with 24μM Mitomycin-C (Sigma) for 1h to prevent cell-proliferation. At day-2, $1x10^6$ hiPSC-CMs (either control or SQTS) were seeded on top of the CoChR-HEK monolayer to generate the co-cultures. The culture medium was supplemented with 5μM Blebbistatin (B0560-5MG, Sigma) to prevent vigorous contraction

Optical mapping of the hiPSC-CCSs co-cultures

Co-cultures were loaded with the voltage-sensitive dye Di4-ANBDQBS (22.5µg/ml, acquired from Leslie Loew, University of Connecticut) for 15min at room temperature. Optical mapping was performed using an EM-CCD (Evolve-512 Delta, Photometrics) and a macroscope (Olympus MVX10). The X-Cite Turbo LED-system served as light source. Excitation filter for Di4-ANBDQBS was Chroma ET620/60x and emission filter was Chroma

ET665lp. Micro-Manager software was used for acquisition, and the OMProCCD software, a custom-made IDL-based software (generously provided by Prof. Bum-Rak Choi, Brown University) served for analysis.

To analyze the data, light artifact from the optogenetic stimulation were eliminated and replaced by a threshold-based custom-made Matlab algorithm. Optical signals at each pixel were then analyzed to measure the local activation time (timing of the maximal dF/dt value) and APD_{80} (the time difference between maximal dF/dt and 80% decay from peak to baseline). These values were used to generate detailed activation and APD_{80} maps.

Immunostaining

Cultures were fixed with 4% paraformaldehyde (Bio-Lab). Blocking was performed by 5% horse serum (Gibco, 1h). An incubation period with primary antibodies was carried out overnight at 4°C. The primary antibodies were used for staining of connexin-43 (1:100; rabbit; Santa Cruz Biotechnology, sc-9059) and α-actinin (1:100; mouse; Sigma-Aldrich, A7811). Samples were washed (x3) with PBS and incubated (1h) with 1:150 diluted secondary antibodies: Cy3 donkey anti-mouse IgG (715-165-151, Jackson Immuno-research laboratories) and Cy5 doneky anti-rabbit IgG (711-175-152, Jackson). Antibodies were diluted in PBS with 3% horse-serum and 0.1% Triton. Nuclei were stained with DAPI (1:500, Sigma, D9564). Zeiss LSM-710 laser-scanning confocal microscope (Zeiss) was used for imaging.

Statistical analysis

Statistical analysis was performed using GraphPad Prism software, except for the Cochran's Q test for binary data that was performed on SPSS. Data were presented as mean±SEM. Differences between the different groups were compared using unpaired student t-test. For studies comparing measurements from the same cells at baseline (darkness) and following optogenetic illuminations, we used the paired student t-test. For studies involving multiple

comparisons of one or two independent variables, either one-way or two-way ANOVA was performed respectively, followed by either post-hoc Tukey or Dunnett (for multiple comparisons with the same control group) tests. To evaluate for potential correlation between the optogenetic stimulation parameters and the resulting changes in APD values, the Pearson correlation coefficient was calculated, and a linear regression model was used. For multiple comparisons of paired binary data (i.e. spiral wave induction rate following different illumination protocols), Cochran's Q test was performed followed by a post-hoc Dunn test. A value of $p<0.05$ was considered statistically significant.

SUPPLEMENTARY FIGURES

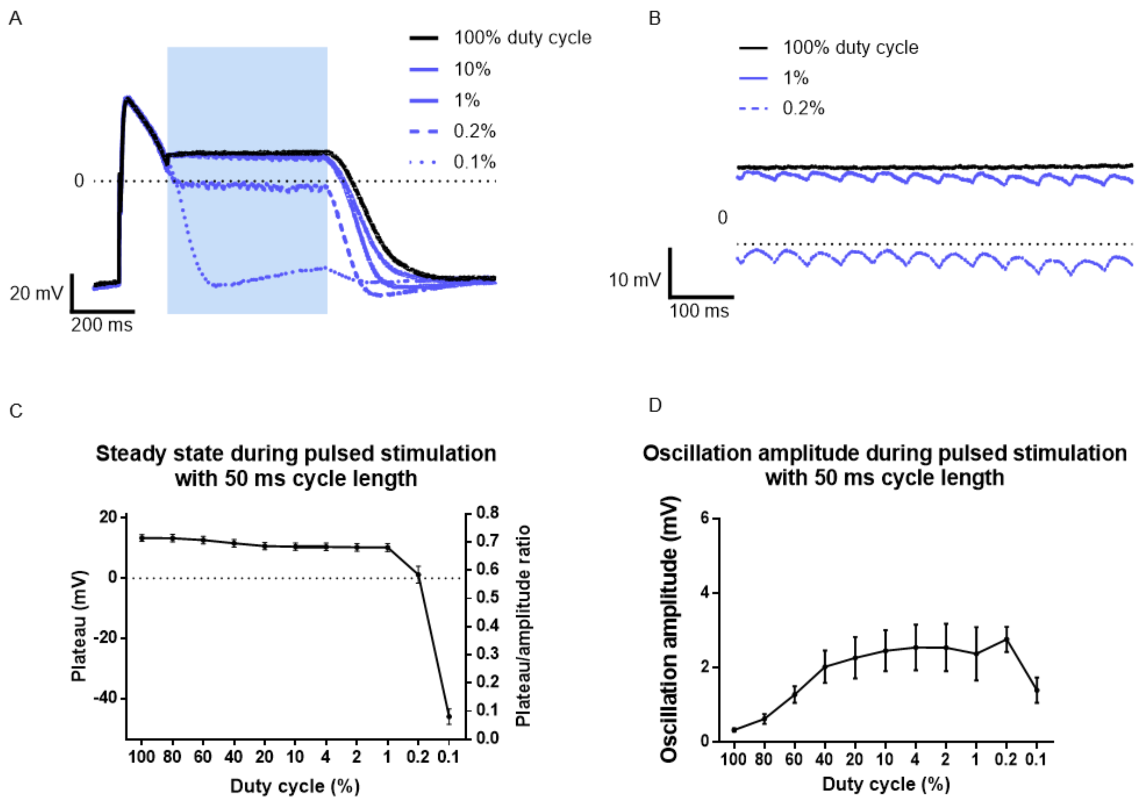

Supplementary Figure 1. Evaluating the effects of different pulsed illumination protocols on action potential properties of ChR2-expressing hiPSC-CMs. [A-B] Representative traces from 5 different experiments, demonstrating the effect of 1 sec-long (20Hz) pulsed illumination delivered using different light pulses types (by varying the pulse duty cycle - representing the percentage of the actual illumination time). **[A]** The result of pulsed illumination given at 100% (continuous illumination), 10%, 1%, 0.2%, and 0.1% duty cycles. Note the similar efficiency of 100%, 10%, and 1% duty cycles. **[B]** Higher resolution images of the traces from panel A, demonstrating the voltage oscillations caused by the high frequency pulsed illumination. **[C-D]** Statistical analysis of the effects of the different illumination patterns on the membrane potential. Illumination pulses (20Hz) were administered at different duty cycles (n=5). **[C]** Analysis of the measured membrane potential during the last 500ms of each pulsed illumination protocol, after a plateau was reached. For every measurement, the corresponding ratio between the plateau value and the AP amplitude is also presented. **[D]** Quantification of the oscillation amplitude caused by the different illumination pulses protocols during the optically induced plateau phase.

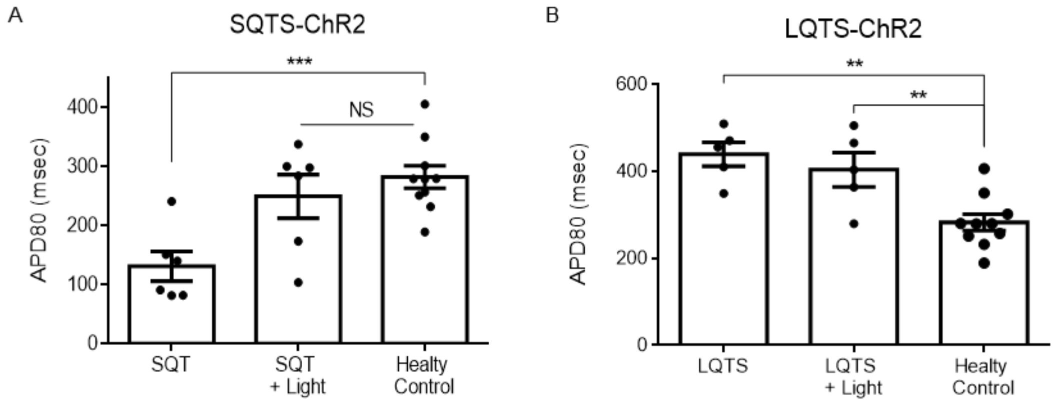

Supplementary Figure 2. Comparison of APD_{80} values between healthy-control hiPSC-CMs and untreated and optogenetically-treated ChR2-expressing SQTS or LQTS hiPSC-CMs.

[A] Comparison of APD_{80} values derived from both untreated and optogenetically-treated (250ms-long optical stimulus, onset-80ms) ChR2-expressing SQTS hiPSC-CMs as compared to heathy-control hiPSC-CMs. Comparisons to the control group were made using one-way ANOVA followed by post-hoc Dunnett's tests. Notice that the SQTS-hiPSC-CMs had significantly (***$p<0.001$) shorter APD_{80} values than healthy-control hiPSC-CMs, whereas optogenetically-treated SQTS-hiPSC-CMs displayed APD_{80} values that were not statistically different (p=NS) from healthy-control cells (n=10 and 6 for healthy and SQTS hiPSC-CMs respectively).

[B] Comparison of APD_{80} values derived from both untreated and optogenetically-treated (onset-40ms, duration-100ms) ChR2-expressing LQTS hiPSC-CMs as compared to heathy-control hiPSC-CMs. Comparisons to the control group were made using one-way ANOVA followed by post-hoc Dunnett's tests (n=10 and 5 for the healthy-control and LQTS hiPSC-CMs respectively). Note that the LQTS-hiPSC-CMs displayed significantly (**$p<0.01$) longer APD_{80} values than healthy-control hiPSC-CMs. Interestingly, the degree of APD_{80} shortening achieved in the optogenetic treatment group was not sufficient to reach APD_{80} values of the healthy-control hiPSC-CMs as APD_{80} values in this group still differed significantly (**$p<0.01$) from the healthy-control group.

See Figure 3 for additional information.

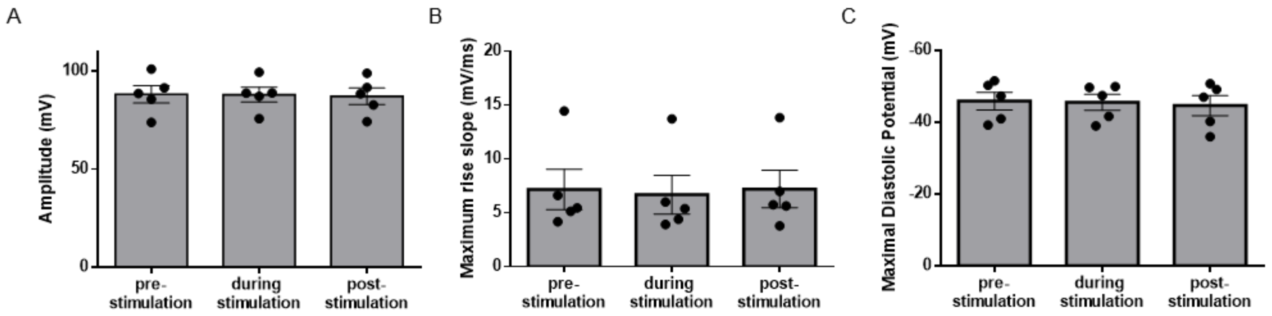

Supplementary Figure 3. Characterizing the effects of the illumination protocol use to activate ChR2 during early phase 2 on AP properties.

Statistical analysis of the change in amplitude (A), maximal rise slope (B), and the maximal diastolic potential (C) before, during and after the illumination protocol. The illumination (onset: 40ms, duration: 100ms) was designed to shorten the APD and prevent EADs in LQTS-hiPSC-CMs. No statistical significance was detected among the groups using a one-way ANOVA test for repeated measurements (n=5).

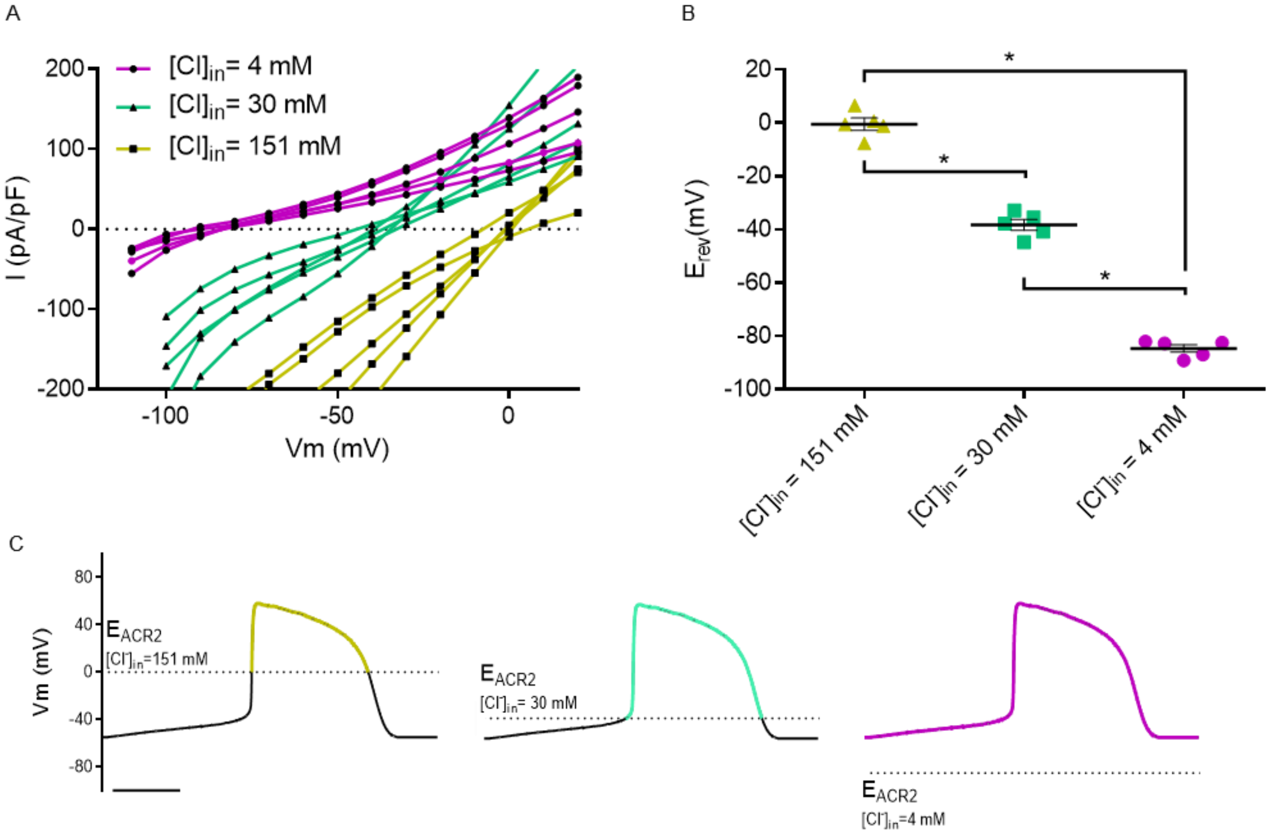

Supplementary Figure 4. Evaluation of ACR2 photocurrents properties and reversal potential in HEK cells.

[A] Virally transduced ACR2-expressing HEK293 cells were subjected to whole-cell voltage-clamp experiments. The stimulation protocol included 1sec-long voltage steps ranging from -110mV to 60mV with 10mV increments. Optical stimulation (1.3mW/mm^2, 470nm) was applied during the second half of each voltage step. The experiments were repeated for 3 different intracellular [Cl$^-$] concentrations [4mM (purple), 30mM (green) and 151mM (yellow)] with a fixed extracellular [Cl$^-$] level (151mM) in all studies. Shown are the resulting I-V curves for the 15 cells studied (5 cells for each of the 3 conditions).

[B] Differences in the measured ACR2 reversal potential (E_{ACR2}) between the three conditions when extracellular Cl$^-$ levels remained fixed (151mM). To measure E_{ACR2}, a linear regression model was generated using the 5 voltage steps closest to the x axis intercept. The average measured E_{ACR2} values were 0mV, -38mV, and -85mV when intracellular Cl$^-$ concentration [Cl$^-$]$_{in}$ were 151mM, 30mM, and 4mM respectively (n=5 for each group, *P<0.001).

[C] A scheme depicting the time-window during the AP, in the three different conditions (colored portion of the APs' tracings), where light-induced ACR2 activation will produce hyperpolarizing photocurrents. Scale bar: 200ms.

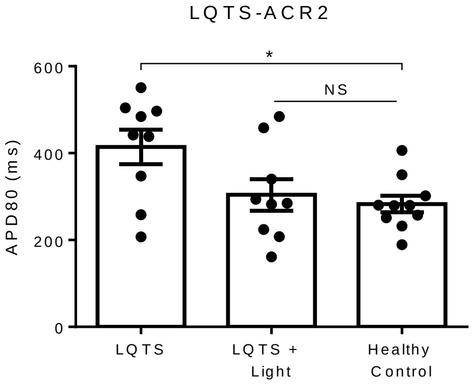

Supplementary Figure 5. Comparison of APD_{80} values between healthy-control hiPSC-CMs and untreated and optogenetically-treated ACR2-expressing LQTS-hiPSC-CMs. Comparison of APD_{80} values derived from both untreated and optogenetically-treated (50ms-long optical stimulus, onset-100ms) ACR2-expressing LQTS hiPSC-CMs as compared to heathy-control hiPSC-CMs. Comparisons to the healthy-control group were made using one-way ANOVA followed by post-hoc Dunnett's tests. Notice that the LQTS-hiPSC-CMs had significantly (*p<0.05) longer APD_{80} values than healthy-control hiPSC-CMs, whereas optogenetically-treated LQTS-hiPSC-CMs displayed APD_{80} values that were not statistically different (p=NS) from healthy-control cells (n=10 and 9 for healthy and LQTS hiPSC-CMs, respectively).

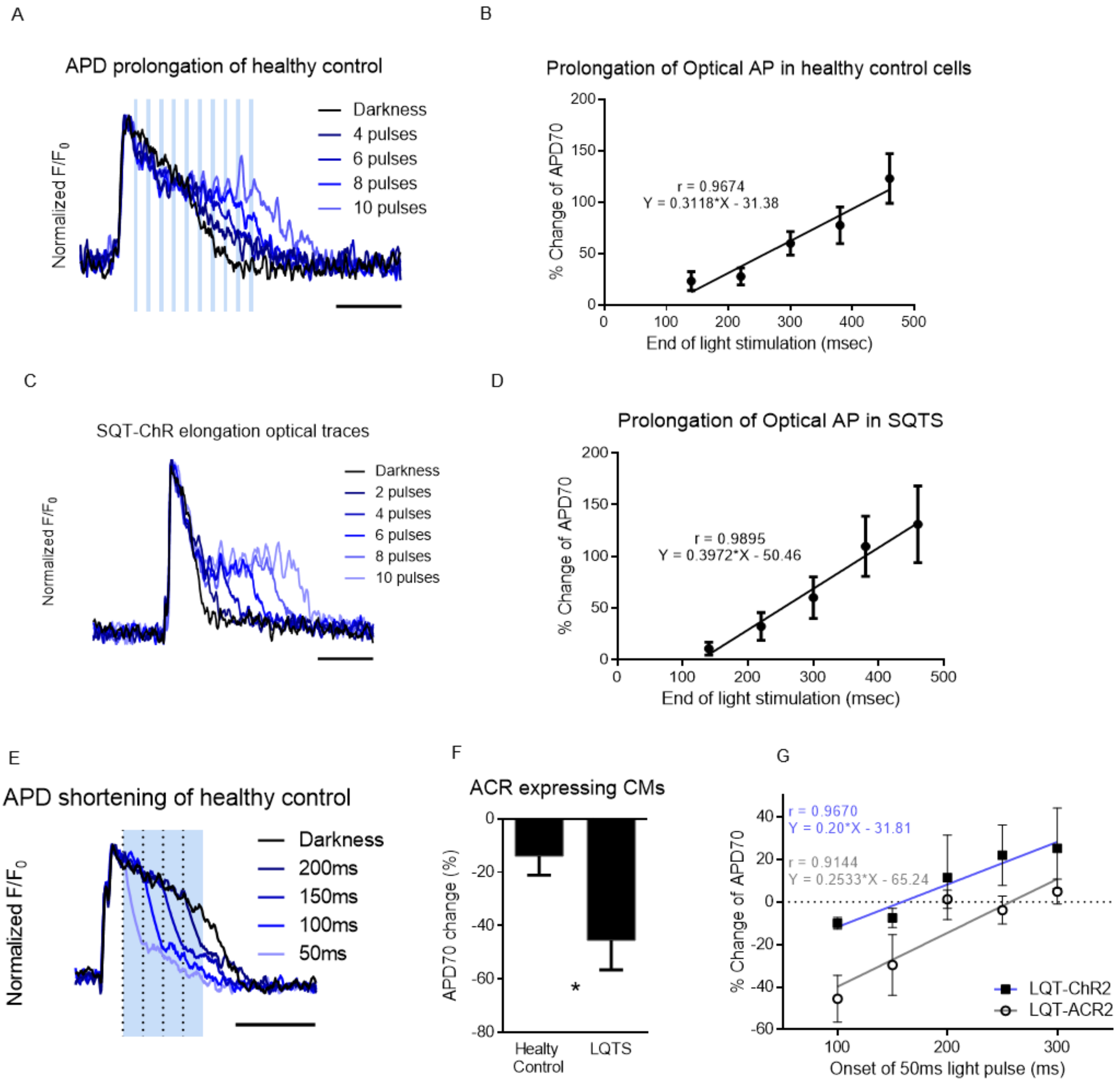
A
APD prolongation of healthy control
Normalized F/F0
Darkness
4 pulses
6 pulses
8 pulses
10 pulses
B
Prolongation of Optical AP in healthy control cells
% Change of APD70
r = 0.9674
Y = 0.3118*X - 31.38
End of light stimulation (msec)
C
SQT-ChR elongation optical traces
Normalized F/F0
Darkness
2 pulses
4 pulses
6 pulses
8 pulses
10 pulses
D
Prolongation of Optical AP in SQTS
% Change of APD70
r = 0.9895
Y = 0.3972*X - 50.46
End of light stimulation (msec)
E
APD shortening of healthy control
Normalized F/F0
Darkness
200ms
150ms
100ms
50ms
F
ACR expressing CMs
APD70 change (%)
Healty Control
LQTS
G
r = 0.9670
Y = 0.20*X - 31.81
r = 0.9144
Y = 0.2533*X - 65.24
% Change of APD70
LQT-ChR2
LQT-ACR2
Onset of 50ms light pulse (ms)

Supplementary Figure 6. Optical monitoring of AP properties during optogenetic stimulation. The hiPSC-CMs were loaded with the voltage-sensitive dye FluoVolt, paced at 1Hz and monitored using the line-scan mode to derive the optical APs. Scale bars: 200ms.
[A-D] Optogenetic prolongation of the recorded optical APs in ChR2-expressing healthy-control **(A-B)** and SQTS **(C-D)** hiPSC-CMs. Shown are representative optically-derived AP signals from a healthy-control **(A)** and a SQTS **(C)** hiPSC-CM at baseline (during darkness, black tracings) and during optogenetic stimulation (blue tracings). Illumination consisted of a pulsed stimulation protocol (15ms-on, 25ms-off), which was initiated 60ms after the AP onset and its duration varied between 2 to 10 light pulses (80-400ms). Note the significant APD prolongation in the recorded optical tracings **(A,C)** and in the summary plots **(B,D)**, and the excellent correlation between the timing of the end of the optical stimuli and the resulting APD_{70} changes [Pearson correlation coefficients: 0.97 (n=10) and 0.99 (n=12) for healthy-control and SQTS hiPSC-CMs respectively].
[E] Optogenetic shortening of the recorded optical APD in ACR2-expressing healthy-control hiPSC-CMs. Shown are the optical APs recorded at baseline (during darkness, black tracing) and during optogenetic stimulation (blue tracing). The timing of the onset of the 50ms-long optical stimulus varied between 50 to 200ms after AP initiation (electrical pacing). Note that the greatest APD shortening was associated with the earliest delivered optogenetic signals. **[F]** Comparison of the degree of APD shortening (relative changes in the optical APD_{70} values) induced by ACR2 activation (50ms-long stimulus, onset-100ms) in the LQTS vs. healthy-control hiPSC-CM. Note that while APD shortening was robust in both cell types, the degree of shortening was significantly greater (*p<0.05) in the LQTS-hiPSC-CMs (n=8) when compared control cells (n=19).
[G] Plots depicting the relationship between the timing of the onset of the optical stimulus (50ms duration) and the relative degree of APD_{70} shortening in LQTS-hiPSC-CMs expressing either ChR2 or ACR2. Note the significantly greater APD shortening associated with ACR2 activation (n=7, **p<0.01) as compared to ChR2 activation (n=4).

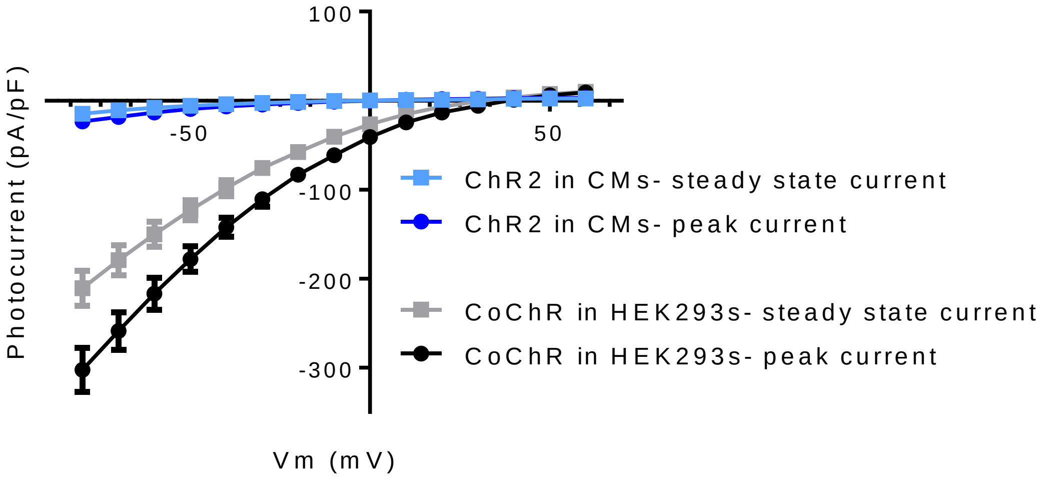

Supplementary Figure 7. Characterization of the ChR2 and CoChR photocurrents in whole-cell patch clamp experiments.
Current-voltage relationship of the peak and steady-state photocurrents are shown for both ChR2 (dark and light blue) and CoChR (black and grey). Currents were measured in the relevant cell type that was used for the single-cell experiments (ChR2 expressing hiPSC-CM) and co-culture tissue model (CoChR-expressing HEK293 cells). The stimulation protocol included voltage steps of 1sec (first 500ms conducted in darkness followed by 500ms of continuous blue-light illumination) from -80mV to 60mV, with 10mV increments. Mean±SEM of peak and steady state currents are plotted (n=7 and n=5 for the ChR2 and the CoChR photocurrents, respectively. Data of ChR2 photocurrents were taken from Fig. 1C.

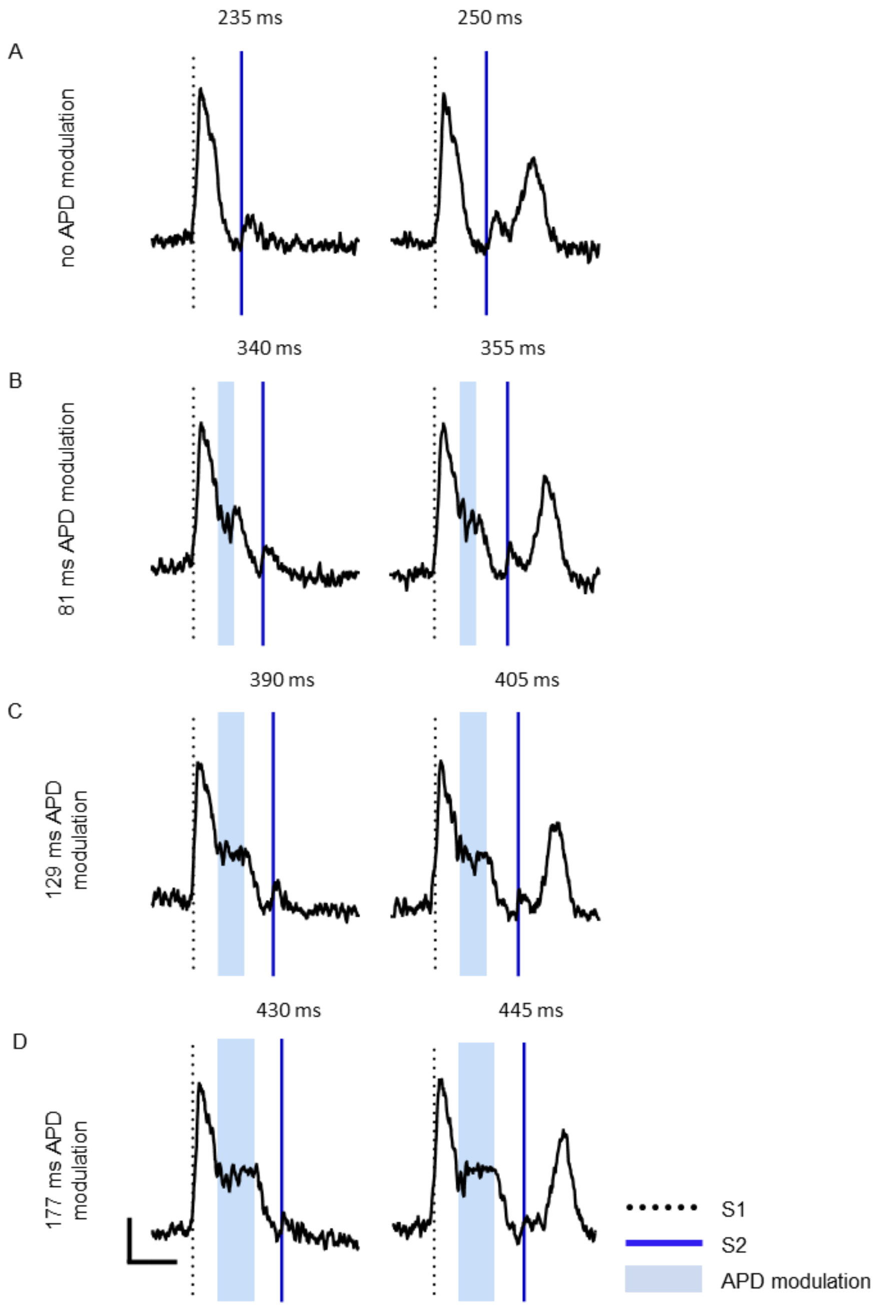

Supplementary Figure 8. Optogenetic APD modulation effects ERP in hiPSC-CCSs
Representative traces of the normalized F/F0 optical signal over time acquired from hiPSC-CCSs. To reveal the ERP, pacing at 1Hz (S1) was followed by a premature stimulus S2. Shown are the optical traces recorded following the S1 and S2 optogenetic signals with the traces on the left-panels showing the longest S1-S2 coupling intervals in which the S2 does not elicit an AP (ERP). The right-panels show optical traces of longer S1-S2 coupling intervals, in which S2 elicits and AP.
ERP was measured at baseline (A) and with different APD modulating illumination durations of 81, 129 and 177 ms (B, C, D, respectively). APD modulating illuminations had onset of 120ms and were delivered as pulsed illuminations. Horizontal scale-bars indicate 200ms.

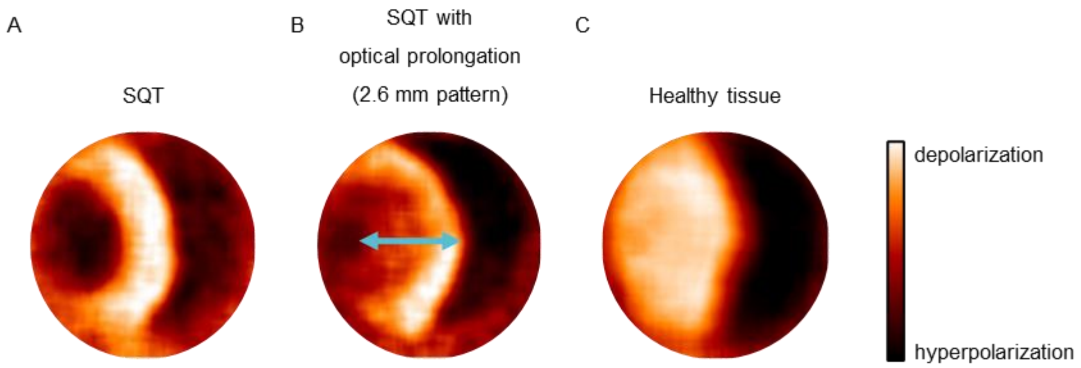

Supplementary Figure 9. Fluorescent maps of propagating waves in hiPSC-CCS. **[A-C]** Representative fluorescent maps of a propagating wave in CCSs, originating from the focal optical pacing site on the left. Shown are wavefronts recorded from a SQTS-hiPSC-CCS (A, taken from Fig. 7C), SQTS-hiPSC-CCS during a dynamic APD modulation illumination protocol with a 2.6mm pattern (B, taken from Fig. 7F and 8A). Identical pacing protocol inducing a propagating wavefront was also induced in a healthy-control hiPSC-CCS (C). Notice that despite the visible repolarization at the tail of the propagating waves in A and B, the depolarized area in the healthy hiPSC-CCS covers the entire left half of the tissue, and therefore has a longer wavelength than A and B.

Supplementary Table 1. Pipette solutions for patch-clamp experiments (concentrations in mM).

	ChR2 experiments	ACR2 experiments (4mM Cl$^-$)	ACR2 I-V curves		
			4mM Cl$^-$	30mM Cl$^-$	151mM Cl$^-$
KCl	120	-	-	26	147
K-Aspartate	-	110	147	121	-
$MgCl_2$	1	1	1	1	1
Mg-ATP	3	-	-	-	-
K_2ATP	-	5	5	5	5
$CaCl_2$	-	1	1	1	1
HEPES	10	10	10	10	10
EGTA	10	11	11	11	11
Related Fig.	Fig. 1-3, 5	Fig. 4, 5	Supplementary Fig. 3~~1~~		

Supplementary Movie Legends

Supplementary Movie 1. Continuous illumination supresses excitability in ChR2-expressing hiPSC-CMs.

The ChR2-expressing hiPSC-CMs were electrically paced at 1 Hz leading to visible contractions at baseline, as the result of AP generation. Notice that prolonged blue-light illumination leads to complete suppression of contraction despite the continuation of electrical pacing. As discussed this suppression of excitability results from clamping of the membrane potential to depolarized values by the continuous light-induced ChR2 activation. Following termination of illumination the cells regain their excitability (responsiveness to the electrical pacing) as manifested by the resumption of contractions.

Supplementary Movie 2. Continuous illumination supresses excitability in ACR2-expressing hiPSC-CMs.

Similarly to Supplementary Movie 1**,** hiPSC-CMs expressing ACR2 were exposed to prolonged blue light illumination (seen as increased brightness) while they were electrically paced at 1 Hz. Cell contraction is visible before and after illumination, but is supressed by the optical stimulus. As discussed, this effect stems from the prolonged light-induced ACR2 activation that clamps the membrane potential to hyperpolarized values, thereby supressing excitability.

Supplementary Movie 3. Development of a dynamically-patterned optogenetic APD-modulation strategy for the prevention of reentrant arrhythmias in SQTS-hiPSC-CCSs.

Patient-specific SQTS-hiPSC-CCSs were seeded on top of a layer of CoChR-expressing HEK293 cells. The cross-field and APD modulating optogenetic stimulation protocols are described in Fig.2. Shown are dynamic displays (videos) depicting the results of the optical

mapping. **(a**) Optical cross-field stimulation induces spiral wave when no APD modulation was applied. **(b)** The initiation of a spiral wave as a result from a similar stimulation protocol as in (a) was prevented when APD modulation protocol was used. This effect stems from augmented refractoriness that inhibits re-excitation of the tissue by the premature beat S2.

References:

1. Itzhaki I, Maizels L, Huber I, Zwi-Dantsis L, et al. Modelling the long qt syndrome with induced pluripotent stem cells. *Nature*. 2011;471:225-229
2. Shinnawi R, Shaheen N, Huber I, Shiti A, et al. Modeling reentry in the short qt syndrome with human-induced pluripotent stem cell-derived cardiac cell sheets. *J Am Coll Cardiol*. 2019;73:2310-2324
3. Burridge PW, Matsa E, Shukla P, Lin ZC, et al. Chemically defined generation of human cardiomyocytes. 2014;11:855-860
4. Shinnawi R, Huber I, Maizels L, Shaheen N, et al. Monitoring human-induced pluripotent stem cell-derived cardiomyocytes with genetically encoded calcium and voltage fluorescent reporters. *Stem Cell Reports*. 2015;5:582-596
